# A fuzzy logic-based secure hierarchical routing scheme using firefly algorithm in Internet of Things for healthcare

**DOI:** 10.1038/s41598-023-38203-9

**Published:** 2023-07-08

**Authors:** Mehdi Hosseinzadeh, Joon Yoo, Saqib Ali, Jan Lansky, Stanislava Mildeova, Mohammad Sadegh Yousefpoor, Omed Hassan Ahmed, Amir Masoud Rahmani, Lilia Tightiz

**Affiliations:** 1 Institute of Research and Development, Duy Tan University, Da Nang, Vietnam; 2 School of Medicine and Pharmacy, Duy Tan University, Da Nang, Vietnam; 3 School of Computing, Gachon University, 1342 Seongnamdaero, Seongnam, 13120 South Korea; 4 Department of Information Systems, College of Economics and Political Science, Sultan Qaboos University, Al Khoudh, Muscat, Oman; 5 Department of Computer Science and Mathematics, Faculty of Economic Studies, University of Finance and Administration, Prague, Czech Republic; 6 Department of Computer Engineering, Dezful Branch, Islamic Azad University, Dezful, Iran; 7 Department of Information Technology, University of Human Development, Sulaymaniyah, Iraq; 8 Future Technology Research Center, National Yunlin University of Science and Technology, Yunlin, Taiwan

**Keywords:** Health care, Medical research

## Abstract

The Internet of Things (IoT) is a universal network to supervise the physical world through sensors installed on different devices. The network can improve many areas, including healthcare because IoT technology has the potential to reduce pressure caused by aging and chronic diseases on healthcare systems. For this reason, researchers attempt to solve the challenges of this technology in healthcare. In this paper, a fuzzy logic-based secure hierarchical routing scheme using the firefly algorithm (FSRF) is presented for IoT-based healthcare systems. FSRF comprises three main frameworks: fuzzy trust framework, firefly algorithm-based clustering framework, and inter-cluster routing framework. A fuzzy logic-based trust framework is responsible for evaluating the trust of IoT devices on the network. This framework identifies and prevents routing attacks like black hole, flooding, wormhole, sinkhole, and selective forwarding. Moreover, FSRF supports a clustering framework based on the firefly algorithm. It presents a fitness function that evaluates the chance of IoT devices to be cluster head nodes. The design of this function is based on trust level, residual energy, hop count, communication radius, and centrality. Also, FSRF involves an on-demand routing framework to decide on reliable and energy-efficient paths that can send the data to the destination faster. Finally, FSRF is compared to the energy-efficient multi-level secure routing protocol (EEMSR) and the enhanced balanced energy-efficient network-integrated super heterogeneous (E-BEENISH) routing method based on network lifetime, energy stored in IoT devices, and packet delivery rate (PDR). These results prove that FSRF improves network longevity by 10.34% and 56.35% and the energy stored in the nodes by 10.79% and 28.51% compared to EEMSR and E-BEENISH, respectively. However, FSRF is weaker than EEMSR in terms of security. Furthermore, PDR in this method has dropped slightly (almost 1.4%) compared to that in EEMSR.

## Introduction

**Figure 1:**
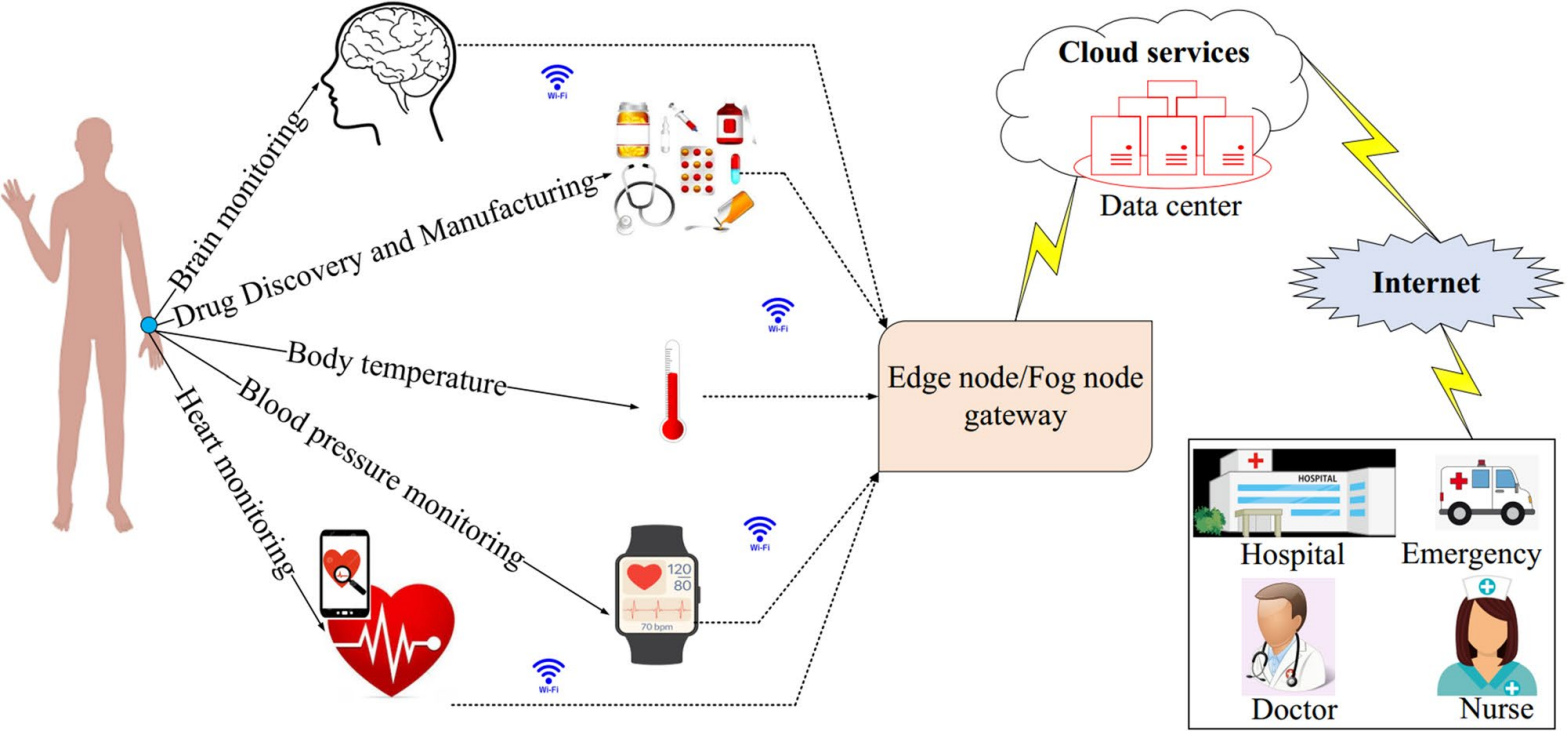
IoT applications in smart healthcare.

The Internet of Things (IoT) is a new platform for creating global communications between billions of devices around the world. IoT and wireless sensor networks (WSNs) are heavily connected to each other because sensors installed on physical objects sense and collect data from the environment, and then process and send it to the base station (BS)^1,2^. Therefore, sensors are important and vital elements in IoT. A WSN-based IoT network is made up of small sensors that measure the environment and collaborate with each other to gather information about the environmental status and send it to the BS or sink node^3,4^. IoT technology can be used in a variety of applications, including healthcare and elderly care. Smart healthcare is an important aspect of human life around the world, and it is expected that the technology will earn several billion dollars in the near future^5,6^. Unfortunately, the continuous aging of the population and chronic diseases have pressurized modern healthcare systems and increased demands for hospital beds, doctors, and nurses^7,8^. For this reason, it is necessary to present a solution to reduce pressure on healthcare systems as well as provide high-quality services to patients. COVID-19 has recently revealed the importance of rapid, comprehensive, and accurate electronic healthcare, including medical and physiological data to detect coronavirus accurately^9,10^. Thus, the use of emerging technologies like IoT in healthcare systems help identify patients and provide conditions for supervising disease treatment and obtaining new evaluations simultaneously. Furthermore, this technology can act as a potential solution to reduce pressure on healthcare systems^11,12^. Figure 1 shows IoT applications in smart healthcare.

The aim of healthcare monitoring is to track the patient’s body parameters and provide fixed and reliable data to physicians or medical teams to better diagnose diseases. This approach will be particularly a great help to patients and elderly users when needing medical services in an unexpected and dangerous situation^13,14^. In smart healthcare, IoT sensors are mainly designed at low cost, low energy consumption, ease of setting up, and stable connectivity. They are responsible for collecting and processing vital data such as electrocardiogram (ECG), oxygen blood saturation, blood pressure (BP), heart rate, blood sugar, pulse rate, brain activity, temperature, and humidity^15,16^. WSN-based IoT networks face energy challenge due to the presence of sensor nodes with limited sources, especially energy. Therefore, obtaining the longest network lifetime is of great significance. Clustering is a successful solution to designing energy-efficient routing schemes because it increases scalability and maintains bandwidth^17,18^. In a clustered network, a sensor node will locally communicate with its cluster head node (CH). In this network, communication with BS is done only through CHs. On the other hand, wearable healthcare applications distribute personal and private data. In this case, security threats such as denial of services (DoS) attacks are carried out by Internet hackers^19,20^. Therefore, hostile nodes obtain and analyze medical data. For this reason, security requirements such as privacy and data integrity should be provided against invaders. In this situation, it is important to create a secure connection between IoT nodes^21,22^. Given that IoT includes heterogeneous devices, security must be guaranteed even for simple devices such as sensors. Therefore, it is important to have a trust framework that prevents the choice of high-risk nodes as intermediate nodes in the routing path. Designing secure and energy-efficient routing protocols to secure data transfer to IoT health devices is a challenging task^23,24^.

In this paper, a fuzzy secure hierarchical routing scheme based on the firefly algorithm (FSRF) is suggested for WSN-based IoT networks. FSRF seeks to achieve two goals, namely improving network security and increasing energy efficiency. Note that security and energy consumption are inversely related to each other because powerful security frameworks usually consume a lot of energy. To solve this challenge in IoT, a secure energy-efficient routing approach must be designed to consider both energy efficiency and trust levels in different phases. FSRF consists of three main frameworks: fuzzy trust framework, firefly algorithm-based clustering framework, and inter-cluster routing framework. The main contributions of this paper are as follows:

- In FSRF, a fuzzy theory-based trust framework is offered to evaluate the trust of IoT nodes and counteract cybersecurity attacks against IoT networks. This framework must be able to detect and prevent various attacks such as black hole, flooding, wormhole, sink hole, and grey hole. Four scales, namely the packet delivery ratio (PDR), packet transfer frequency (PTF), packet reception frequency (PRF), and the consumed energy ratio (ECR) are considered to design this trust framework.
- In FSRF, a clustering method based on the firefly algorithm is offered to lower the energy consumed by IoT nodes, communication overhead, and congestion on the network. In this clustering technique, a new objective function is suggested. This function helps improve network security in the clustering process because it includes the trust level of IoT nodes. Also, paying attention to the energy level and the number of hops between CHs and the base station in this objective function has improved energy efficiency in this method.
- In FSRF, a routing method is offered to find reliable and energy-efficient routes between nodes to reduce the risk of forming fake and unsafe paths in the network because in this scheme, reliable nodes participate in the route discovery process and hostile nodes are not allowed to cooperate in this process.
- In this paper, FSRF is compared with EEMSR and E-BEENISH with regard to network longevity, energy consumption, and packet delivery ratio. This comparison shows that FSRF well guarantees energy efficiency in the network because it increases the network longevity by 10.34% and 56.35% and the energy level stored in the nodes by 10.79% and 28.51% compared to EEMSR and E-BEENISH, respectively. However, FSRF is weaker in terms of security than EEMSR and it has less PDR (almost 1.4%) than EEMSR.

The structure of the paper is as follows: “Related works” section examines some research on cyber security attacks on IoT and their countermeasures. In “Base concepts” section, the main concepts used in the FSRF, namely the firefly algorithm (FA) and fuzzy logic are expressed. “System model” section includes the network model, the energy model, and the attack model. “The proposed method” section has stated various steps of FSRF. Simulation and evaluation results are presented in “Simulation and result evaluation” section. The conclusions obtained from this paper are described in “Conclusion” section.

## Related works

In^25^, a security framework for routing is provided to prevent cyber security attacks in the Industrial Internet of things (IIoT). For designing this framework, the authors have benefited from different technologies like software-defined networking (SDN), network function virtualization (NFV), and blockchain. The designed framework is flexible, programmable, and secure. Moreover, a three-level SDN/NFV framework is employed in each domain to control and plan desired forwarding devices when calculating optimal routing policies. On the other hand, SDN controllers employ a blockchain framework to build a reliable environment. Next, a secure routing scheme based on this framework is introduced to support node authentication and behavior authentication in the network. The simulation process is done on the OMNET++ platform. The results show that the proposed system is better than other schemes with regard to scalability and stability when occurring attacks.

In^26^, a reliable and efficient route selection solution called REERS is offered to get better energy efficiency and lower delay in IoT applications. Initially, REERS employs a clustered data aggregation model that regards energy levels for selecting cluster heads among IoT devices. Next, CHs collect the sensed data and delete duplicated information. Thereafter, various routes are found to the destination for transferring aggregated data packets. Finally, these packets will be directed with the lowest consumed energy, less hop count, and less lost data. The experimental results show that REERS optimizes delay, network longevity, as well as throughput, and PDR.

In^27^, the authors have introduced a blockchain-based lightweight authentication structure to check the validity of ordinary sensors. Note that IoT sensors have a short lifetime due to energy restrictions, thus they require a small validity value in blockchain to obtain a lightweight authentication structure. The network controller employs a genetic algorithm-based software to calculate paths. In addition, an on-demand routing technique is applied to optimize the energy used by nodes. The authors have suggested a path-checking framework to investigate the existence of malicious nodes. Furthermore, a novel structure is introduced to limit the activity of the hostile nodes. A list of hostile nodes is stored in the blockchain. This list is employed by the path-checking structure. The experimental results indicate the successful performance of this method with regard to consumed energy and the detection rate of hostile nodes.

In^28^, a routing technique based on the shuffled frog-leaping algorithm (SFLA) called RISA is provided for the Internet of Things. This technique employs SFLA to pick out a content-based path from source to destination. Content-based routing decreases the number of transmitted data packets and redundancy through data aggregation. This operation has a great impact on maintaining network resources. When a data packet moves from source to destination, the shortest and most optimized path must be selected to minimize energy consumption. RISA regards energy efficiency and consequently improves network longevity since it applies an appropriate data aggregation technique. Simulation results in MATLAB software show that RISA can optimize energy consumption, network longevity, throughput, and PDR.

In^29^, a Harris Hawks Optimization (HHO)-based reliable data dissemination technique called RDDI is suggested for IoT. This secure data dissemination framework offers a fuzzy hierarchical network model for WSN-based IoT networks. RDDI detects attacks and supervises information exchanged between nodes. It builds the data transfer process based on the energy and geographic location of nodes to improve the routing capability. Additionally, it employs a fuzzy clustering structure to pick out a trusted path. The authors evaluated RDDI with regard to five criteria, including reliability, end-to-end delay, consumed energy, computational overhead, and packet-sending distance in different multi-cluster scenarios. The experimental results emphasize the successful performance of RDDI in comparison with other approaches with regard to energy consumption, reliability, end-to-end delay, and computational overhead.

In^18^, a tree-based secure routing scheme supported by a dragonfly algorithm called CTSRD for IoT-based smart agriculture. It employs a decentralized and light trust structure called W-Trust. This structure regards a punishment coefficient to decrease the trust of hostile nodes. In contrast, it grows the trust of the valid nodes according to a growth coefficient. Furthermore, it makes a trusted clustering framework (T-Clustering). In this framework, CHs are selected from valid nodes. Eventually, CTSRD builds an inter-cluster routing tree inspired by the dragonfly algorithm named DA-Tree, which is safe, sustainable, and optimal and balances the energy used in the network and extend the longevity of the network. The experimental results emphasize that CTSRD is able to distribute consumed energy uniformly in comparison with other approaches and consequently gets better network longevity. However, PDR in this scheme is low.

In^30^, a multi-level trusted energy-efficient routing scheme called EEMSR in IoT. The authors have used a clustering technique in this method since EEMSR is a effective solution in terms of energy consumption and scalability. In EEMSR, the analytic hierarchy process (AHP) is employed to forecast accurate weight coefficients in the normalization operation. Furthermore, an enhanced genetic algorithm (GA) is suggested to get the best performance and decide on intermediate nodes in multi-hop paths. This enhanced GA lowers the consumed energy in the network and counteracts the weaknesses of GA in the routing process. In addition, a multi-trust framework has been employed to defend against different attacks on the network. This framework computes the trust coefficient in the clustering and routing process. This coefficient is obtained from three trust values, including data perception, data fusion, and communication trust. EEMSR has also focused on both energy efficiency and security. The experimental results emphasize the successful performance of EEMSR compared to other schemes.

In^31^, an energy-efficient routing technique named E-BEENISH is presented for heterogeneous WSNs. It analyzes the energy used in inter-cluster and intra-cluster communications to balance energy consumption. E-BEENISH regards a weighted probability for each node when choosing CHs. This probability relies on remaining energy and the distance from the sink node to the desired node. E-BEENISH introduces a simple algorithm that considers the distance between the desired node and BS to overcome the threshold settings in BEENISH. The authors also studied the effect of the heterogeneity of sensor nodes in terms of energy used in the network. Simulation results show that E-BEENISH gets better network longevity in comparison to other clustering protocols.

In WSN-based IoT networks, sensor nodes include various constraints, especially energy, memory, and computing power. These constraints have faced challenges such as shortening the network lifetime and increasing the lost data packets. Therefore, the necessity of a hierarchical routing method in these networks is increasingly evident because clustering is a successful solution for designing energy-efficient routing methods. According to research works studied in this section and Table 1, it can be seen that in recent years, many hierarchical routing methods, for example, REERS^26^ and E-BEENISH^31^ have been provided in wireless sensor networks to manage energy consumption in these networks. However, these methods do not pay attention to the security issue and, if there are hostile nodes on the network, they will face weak performance. Note that sensor nodes can be deployed in a hostile environment and may be easily captured by attackers, which prevent the proper network performance by doing hostile operations. In this condition, it is important to create a secure connection between IoT nodes. Hence, many researchers focus on powerful security methods, such as Cao et al.^25^ and Abbas et al.^27^. These methods are often not suitable for WSNs because they do not pay attention to the energy constraints of the nodes, which can reduce network longevity and lose their normal performance. This shows that the design of secure and energy-efficient routing protocols is a very important issue. Among the methods studied in this paper, some researchers, for example, RDDI^29^, CTSRD^18^, and EEMSR^30^ have taken into account both energy efficiency and security. However, research on hierarchical and secure routing methods is still known as an important research gap, which requires further investigation and analysis. In this paper, a fuzzy secure hierarchical routing scheme based on the firefly algorithm (FSRF) is proposed for WSN-based IoT networks. In FSRF, a fuzzy theory-based trust structure is provided to evaluate the trust of IoT nodes and counteract cybersecurity attacks against IoT networks. Furthermore, this method includes a clustering method based on the Firefly algorithm that improves network security in the clustering process because it considers the trust levels of the nodes, and improves energy consumption in the network because it pays attention to the energy level and the number of hops to the base station. FSRF presents a routing method for finding reliable and energy-efficient routes between nodes in the network.

**Table 1:** Comparison of the related works.

Method	Publication year	Security mechanism	Routing technique	Energy efficiency	Strengths	Weakness
Cao et al.^25^	2021	Blockchain	An SDN-based secure routing	$\times$	Designing a flexible, programmable, and secure routing framework	Not paying attention to the energy index in the routing process
REERS^26^	2021	$\times$	Clustering routing method	$\checkmark$	Increasing energy efficiency and reducing delay	Not having a security mechanism
Abbas et al.^27^	2021	Blockchain	A GA-based routing protocol	$\times$	Designing a lightweight authentication structure, ability to accurately detect attacking nodes	Not paying attention to the energy index in the routing process, high execution and transaction costs
RISA^28^	2020	$\times$	A SFLA-based content centric routing method	$\checkmark$	Increasing energy efficiency	Not designing a security mechanism
RDDI^29^	2020	HHO-based watchful node selection process	A hierarchical energy-aware geographic routing based on the fuzzy clustering	$\checkmark$	Enhancing energy efficiency, detecting and isolating malicious nodes	Not evaluating its robustness and efficiency against cybersecurity attacks
CTSRD^18^	2023	A decentralized and light trust structure called W-Trust	A tree-cluster based routing scheme supported by a dragonfly algorithm	$\checkmark$	Considering energy efficiency, using a tree-cluster network topology	Low packet delivery rate (PDR)
EEMSR^30^	2021	A multi-trust framework based on data perception trust, data fusion trust, and communication trust	AHP-based clustering and a GA-based routing protocol	$\checkmark$	Balancing energy consumption in the network, designing a strong security mechanism, detecting malicious nodes	High time complexity
E-BEENISH^31^	2019	$\times$	A clustering routing technique	$\checkmark$	Considering remaining energy and the distance from the sink node for selecting CHs, high scalability	Not designing a security mechanism
FSRF	$\times$	A fuzzy trust mechanism	A clustering routing method based on firefly algorithm	$\checkmark$	High network lifetime, high scalability, considering energy efficiency, using a strong security mechanism	Low packet delivery rate (PDR)

## Base concepts

FSRF employs a nature-inspired optimization algorithm named the firefly algorithm (FA) and fuzzy logic (FL). Therefore, these two techniques are explained in this section.

### Nature-inspired optimization algorithms

These algorithms are rooted in the social behaviors of biological species, for example, birds, fish, ants, and fireflies. In this system, the behaviors of agents that interact locally with their environment have led to the emergence of coherent global patterns. These algorithms have benefited from self-organization, parallel operations, distributed operations, flexibility, and stability^32,33^. For this reason, their applications have gradually expanded to solve many IoT issues, including routing such as^18,28,29^, and^34^. They are used to solve many real-world engineering issues. For example, the firefly algorithm (FA) is a nature-inspired optimization algorithm presented by Yang in 2007^35^. This algorithm has benefited from flexibility in the population. This means that it is scalable. It can perform a relatively large search and corrects the responses in the search process. When getting the optimal solution, FA makes a balance between exploration and exploitation, and eventually reaches an optimal global behavior. These advantages have led to the use of this technique in the proposed method. This algorithm is inspired by the brightness of the fireflies. In the FA algorithm, there are two important issues: light intensity changes and formulation of brightness and attractiveness. It is assumed that the attractiveness of fireflies is proportional to their brightness. Moreover, the brightness is determined by an objective function. In this algorithm, the less-light firefly will be attracted to the high-light firefly^35^.

### Fuzzy logic

Fuzzy logic is a good scheme for mapping from the input space into an output space. It is a precise method based on approximate and inaccurate data. Fuzzy systems (FSs) are defined by fuzzy set theory^36,37^. A fuzzy set is a borderless set, which includes elements with partly membership degree (usually between 0 and 1). The fuzzy system may be less accurate than conventional systems, but more like our everyday experiences as human decisions. The fuzzy inference is the mapping process from a given input to an output using FL. Then, this mapping provides a basis for decision-making. The fuzzy inference comprises membership functions (MFs), fuzzy operators, and IF-THEN rules. Mamdani and Sugeno are two common fuzzy inference systems. A Mamdani system states that the output MFs are fuzzy sets. After aggregating the results, there is a fuzzy set for each output variable, which requires a defuzzifier. A Sugeno system also supports this behavior and is very similar to the Mamdani system. In fact, the first two parts of the fuzzy inference process, the fuzzifier process, and the use of fuzzy operators are similar. The main difference between Mamdani and Sugeno models is that output MFs in Sugeno can be fixed or linear. Whereas, output MFs in Mamdani are nonlinear^36,37^.

Usually, FS has four components: fuzzifier, fuzzy rules, inference engine, and defuzzifier. Fuzzification must categorize numerical scales into fuzzy sets. The knowledge base consists of IF-THEN rules that show linguistic reasoning. An inference engine executes the rules on the fuzzy inputs to get fuzzy results. The fuzzy controller needs the knowledge of an expert or operator experience to determine appropriate control rules and MFs. Fuzzifier can convert crisp data or fuzzy data into appropriate linguistic values through language variables and a variety of MFs, such as triangular, trapezoidal, and Gaussian. MF maps from each element of the input variables to a membership degree between 0 and 1. Triangular functions are usually used in FSs because of their simplicity. Finally, defuzzifier determines how to extract the crisp value from the fuzzy set. The well-known defuzzifier is the centroid, which shows more reliable results than others. The selection of a defuzzifier is very important and has a significant impact on the speed and accuracy of the fuzzy model^38^.

## System model

This section describes different parts of the system model namely the network model, the energy model, and the attack model.

### Network model

**Figure 2:**
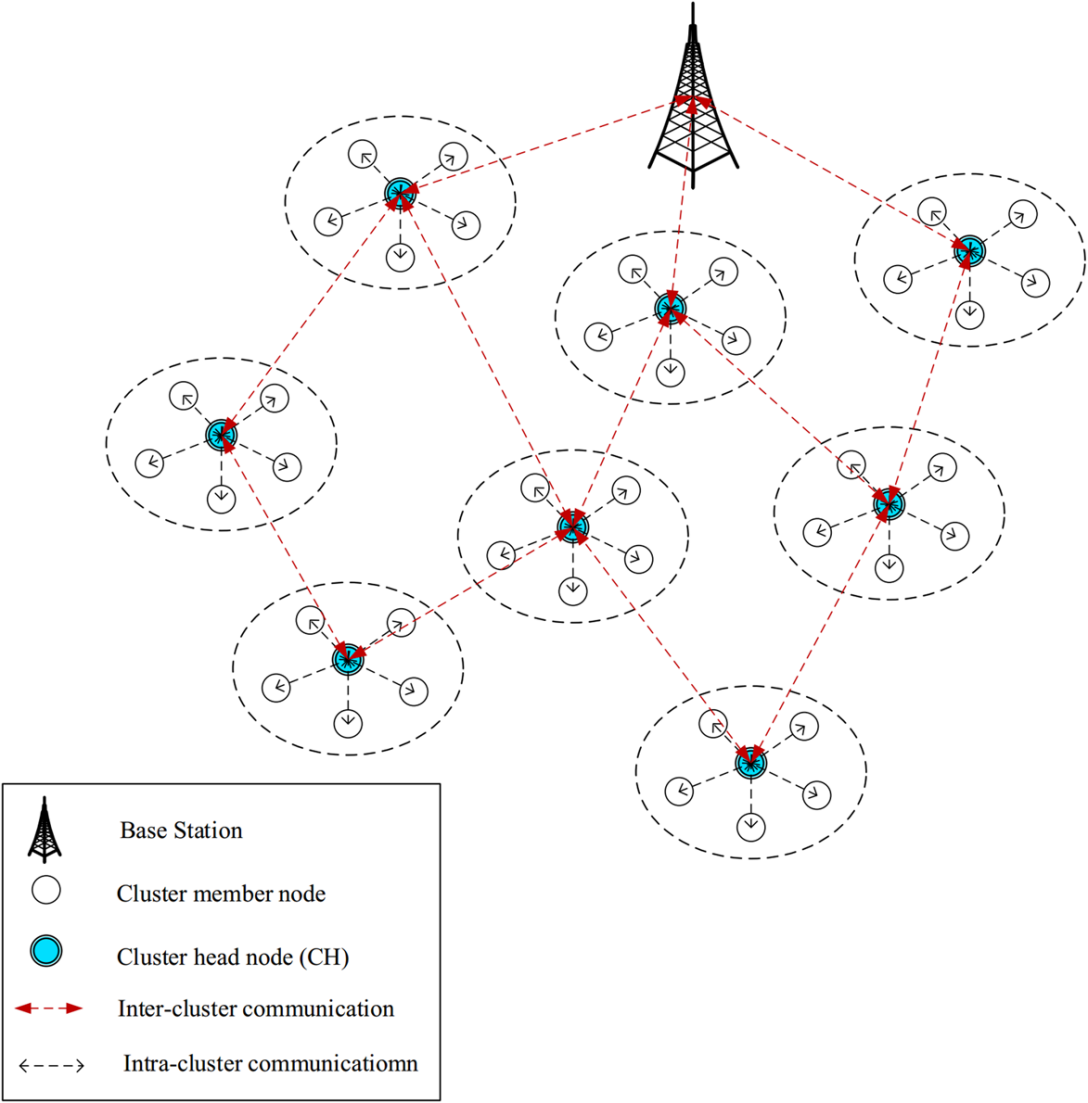
Network model in FSRF.

In FSRF, the network is made up of heterogeneous IoT nodes ($n_{1},n_{2},...,n_{i},...,n_{T}$, so that *T* indicates the total number of nodes in the network) and a base station. The BS has different responsibilities, like data analysis and decision-making about data received from cluster heads. It is a motionless node, and all nodes know its position on the network. A special identifier is employed by each node (i.e. $n_{i}$). The distribution of the nodes in the network is done using a random manner. In FSRF, there is a connection between network nodes and the global positioning system (GPS). Hence, the nodes can obtain their spatial coordinates in the network. FSRF regards heterogeneous nodes, which have different energy levels, computational power, and storage capacity. In FSRF, the nodes are placed in clusters. Each cluster is made up of a cluster head (CH) and a number of cluster members (CMs). CMs have various responsibilities such as sensing the environment and transferring the data to the CH. They perform the intra-cluster data transfer operation using a single-hop manner. In addition, CHs have one important responsibility i.e. gathering data from its CMs and transferring the aggregated data to BS. They perform the inter-cluster data transfer operation using a multi-hop manner to transmit their data to the base station. See the network model in Fig. 2.

### Energy model

The data transfer operation, which means sending and receiving data, is known as the most serious factor of energy consumption in the network. FSRF regards both free space and multi-path models to control how much energy is consumed in the recipient and sender. Note that the energy model used in this paper is similar to the energy radio model proposed by Heinzelman et al.^39^. In this case, the whole energy used in all nodes is equal to their consumption energy when sending and receiving data.

1 $$\begin{aligned} E_{Total}=\sum \limits _{i=1}^{N}{\left( E_{tx}^{i}+E_{rx}^{i}\right) } \end{aligned}$$

Here, $E_{tx}^{i}$ and $E_{rx}^{i}$ express the required energy of ${n_{i}}$ for transferring and getting data, respectively.

When two nodes want to exchange their data with each other. In this case, one of them acts as a transmitter (also called $n_{t}$) and the other node plays the role of a receiver (also called $n_{r}$). Suppose *l* indicates the size of the exchanged data and $d=\sqrt{\left( x_{r}-x_{t}\right) ^{2}+\left( y_{r}-y_{t}\right) ^{2}}$ is the distance from $n_{t}$ to $n_{r}$ where $\left( x_{r},y_{r}\right)$ and $\left( x_{t},y_{t}\right)$ are the spatial coordinates of $n_{r}$ to $n_{t}$, respectively. In this case, Eq. (2) determines how much energy is used by $n_{t}$.

2 $$\begin{aligned} E_{tx}\left( l,d \right) =\left\{ \begin{matrix} l\times {E_{elec}}+l\times {{\varepsilon }_{fs}}\times {d^{2}},\,\,\,\,\,\,d<{d_{0}} \\ l\times {E_{elec}}+l\times {{\varepsilon }_{mp}}\times {d^{4}},\,\,\,\,\,\,d\ge {d_{0}} \\ \end{matrix} \right. \end{aligned}$$

So that $E_{elec}$ is the energy needed for the electrical equipment of $n_{t}$ or $n_{r}$, ${\varepsilon }_{fs}$ represents the amplification factor in the free space, and ${\varepsilon }_{mp}$ indicates the amplification factor in the multi-path space. In Eq. (2), if the distance between $n_{t}$ and $n_{r}$ (i.e. *d*) is shorter than ${d_{0}}$ (i.e. the boundary value), the consumed energy is calculated based on the free-space model (the first line of Eq. (2)); otherwise, it is calculated based on the multipath model (the second line of Eq. (2))^39^. ${d_{0}}$ is obtained from Eq. (3), which demonstrates a boundary condition for the data transfer scheme employed by $n_{t}$ and $n_{r}$.

3 $$\begin{aligned} d_{0}=\sqrt{\frac{{{\varepsilon }_{fs}}}{{\varepsilon _{mp}}}} \end{aligned}$$

Finally, Eq. (4) determines how much energy is used by $n_{r}$:

4 $$\begin{aligned} {E_{rx}}\left( l \right) =l\times {E_{elec}} \end{aligned}$$

### Attack model

IoT employs wireless channels to communicate between the network nodes. Therefore, this network is exposed to serious security harm. The invading nodes can penetrate the network in different ways and launch various attacks on the network. This ruins the normal network performance and affects the secure data transfer operation due to the removal or manipulation of data packets and the energy discharge of the IoT nodes. Therefore, it is necessary that the nodes involved in the process are safe and reliable. FSRF focuses on blackhole, sinkhole, wormhole, selective forwarding, and flooding.

- *Black hole or sinkhole attacks:* In these attacks, when an invading node (black hole or sinkhole) gets a route request from other network nodes, it replies to this message to state that it has a suitable path to the destination, even though this claim is not right. When the requested node obtains this response, it may employ the insecure path, which includes a black hole or sinkhole, for transferring data. In this case, the invading node eliminates all data packets received from the source node.
- *Wormhole attack:* In this attack, two invading nodes build a tunnel and state that they are neighbors (i.e. they are very close together) while this claim may be wrong. High-power nodes may carry out this attack. In this case, they have more resources than normal nodes. When the invading nodes build a forged path, they seek to attract network traffic and declare that the path is very efficient and suitable, and has smaller hops to the BS while it is not fact. After attracting the data traffic of normal nodes, the invading nodes can track their communications, copy and manipulate their data packets.
- *Selective forwarding attack:* This attack, also called grey hole, is an advanced model of black hole attacks. In this attack, the invading node selectively deletes some packets but not all of them. It deletes only packets transmitted to a specific destination or eliminates a special type of packets.
- *Flooding attack:* In this attack, the invading node continually transmits route requests to a specific node. This work leads to the discharge of the target node, its storage space is full. This is because the invading node misuses the fact that some information about the route requests is stored in the memory of the target node. In this case, the target node cannot respond to the legal requests of other node and dies quickly because it loses high energy.

## The proposed method

In this section, a fuzzy secure hierarchical routing scheme based on the firefly algorithm (FSRF) is explained for WSN-based IoT networks. FSRF comprises three main frameworks: fuzzy trust framework, firefly algorithm-based clustering framework, and inter-cluster routing framework.

### Fuzzy trust framework

In FSRF, the fuzzy trust framework is tasked to analyze the reputation of nodes according to their interactive behavior when transferring and receiving data packets. Determining the trust value of the network nodes will be done using a fuzzy trust framework. Algorithm 1 offers a pseudo-code of the fuzzy trust framework. The Mamdani fuzzy system is used to design this framework. It comprises two inputs (i.e. direct trust and indirect trust), an output (i.e. total trust of network nodes), and the rule base. Each IoT node executes this fuzzy framework to characterize the trust value of its neighboring nodes. Additionally, the trust value of the nodes changes dynamically, and their energy decreases, and some of them die. Therefore, IoT nodes must renew the trust values related to their neighboring nodes at certain time intervals.

#### Fuzzy inputs

The proposed fuzzy framework comprises two inputs called direct trust and indirect trust.

- *Direct trust of *${n_{i}}$ *related to* ${n_{j}}$ (${T_{ij}^{direct}}$)*:* $T_{ij}^{direct}$ indicates the urgent trust generated by $n_{i}$ for $n_{j}$. It is acquired through the direct connection of $n_{i}$ and $n_{j}$. In FSRF, the direct trust regards the packet delivery ratio (PDR), packet transfer frequency (PTF), packet reception frequency (PRF), and the consumed energy ratio (ECR).
  - $PDR_{j}$ means a packet reception rate corresponding to $n_{j}$. It expresses the ratio of packets received by $n_{j}$ to all data packets sent to this node. Note that a high PDR confirms the successful performance of $n_{j}$ and shows its reliability. However, if $n_{j}$ does not experiences a suitable PDR, it means that $n_{j}$ has high missing data. In this case, $n_{j}$ may be an invader. This increases the probability of attacks such as black holes, sink hole, and grey hole. Hence, the trust value corresponding to $n_{j}$ decreases. $PDR_{j}$ is calculated through Eq. (5).
    5 $$\begin{aligned} PDR_{j}=\frac{M_{j}^{received}}{M_{j}^{total}} \end{aligned}$$
    Here, $M_{j}^{received}\left( t \right)$ and $M_{j}^{total}$ represent the number of packets received and sent to $n_{j}$, respectively.
  - $PTF_{j}$ determines how many packets are transferred by $n_{j}$ at the time interval $\left[ t,t+\Delta t\right]$. Note that a high PTF means that $n_{j}$ may be an invader because there is a high likelihood of flooding or wormhole attacks. In this case, the trust value of $n_{j}$ is reduced. $PTF_{j}$ is calculated by Eq. (6).
    6 $$\begin{aligned} PTF_{j}=\frac{M_{j}^{Transferred}}{\Delta t} \end{aligned}$$
    where $M_{j}^{Transferred}$ counts how many packets are transferred at the interval $\left[ t,t+\Delta t\right]$.
  - $PRF_{j}$ determines how many packets are received by $n_{j}$ at the time period $\left[ t,t+\Delta t \right]$. Note that a high $PRF_{j}$ confirms that $n_{j}$ is safe and reliable. However, if $n_{j}$ gets low $PRF_{j}$, $n_{j}$ may be an invader because the probability of attacks such as black hole, sinkhole or gray hole is high. $PRF_{j}$ can be achieved through Eq. (7).
    7 $$\begin{aligned} PRF_{j}=\frac{M_{j}^{Received}}{\Delta t} \end{aligned}$$
    So that $M_{j}^{Received}$ counts the packets received in the interval $\left[ t,t+\Delta t \right]$.
  - $ECR_{j}$ determines how much energy is consumed by $n_{j}$ in the time period $\left[ t,t+\Delta t\right]$. Note that a high $ECR_{j}$ states that $n_{j}$ may be an invader because the probability of a flooding attack is high. $ECR_{j}$ is determined through Eq. (8).
    8 $$\begin{aligned} ECR_{j}=\frac{{E_{j}}\left( t \right) -{E_{j}}\left( t+\Delta t \right) }{\Delta t} \end{aligned}$$
    So, $E_{j}\left( t \right)$ and ${E_{j}}\left( t+\Delta t \right)$ are the residual energy of $n_{j}$ in two times *t* and $t+\Delta t$, respectively.

According to the stated parameters, $T_{ij}^{direct}$ is calculated based on Eq. (9).

9 $$\begin{aligned} T_{ij}^{direct}=\frac{{{\lambda }_{1}}PDR_{j}+{{\lambda }_{2}}PRF_{j}}{{{\lambda }_{3}}PTF_{j}+{{\lambda }_{4}}ECR_{j}} \end{aligned}$$

So, ${\lambda }_{1}$, ${\lambda }_{2}$, ${\lambda }_{3}$, and ${\lambda }_{4}$ are the weight coefficients adjusted in $\left[ 0,1 \right]$ and $\sum \limits _{i=1}^{4}{{{\lambda }_{i}}=1}$. In FSRF, the window mean with exponentially weighted moving average (WMEWMA) is employed to renew $T_{ij}^{direct}$. It applies the window length *w* to consider the historical trust amounts when calculating $T_{ij}^{direct}$. Hence, $n_{i}$ does not only rely on the present amount and uses the set of trust values to better decide on $T_{ij}^{direct}$. As a result, Eq. (10) renews $T_{ij}^{direct}$.

10 $$\begin{aligned} T_{ij}^{direct}\left( l \right) =\left( 1-\beta \right) \frac{\sum \limits _{k=l-w}^{l-1}{T_{ij}^{direct}\left( k \right) }}{w}+\beta T_{ij}^{direct}\left( t \right) \end{aligned}$$

**Figure 3:**
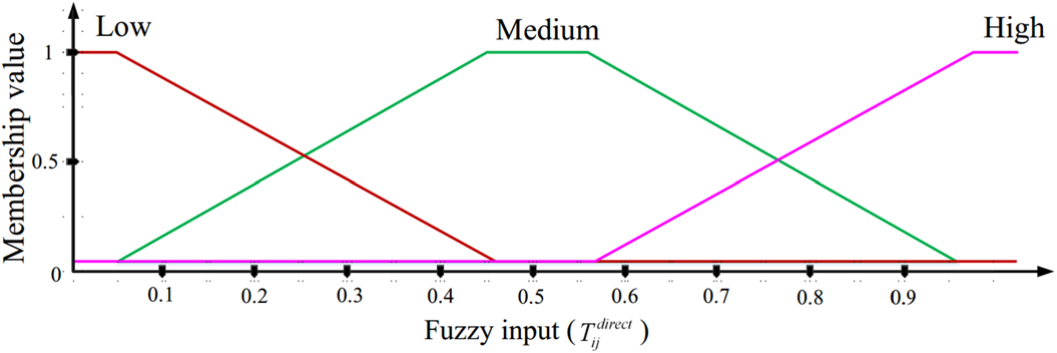
MF related to $T_{ij}^{direct}$.

So that $\beta$ is a coefficient adjusted in $\left[ 0,1 \right]$. Membership function (MF) related to $T_{ij}^{direct}$ is depicted in Fig. 3. $T_{ij}^{direct}$ contains three modes, low, medium, and high.

**Figure 4:**
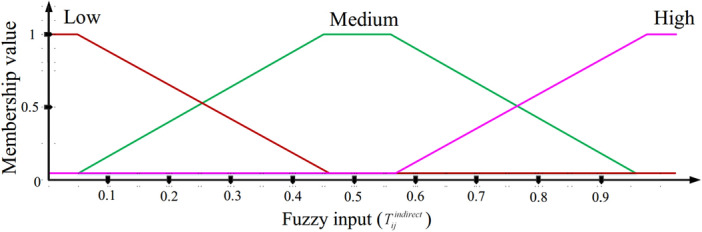
MF of $$T_{ij}^{indirect}$$.

- *Indirect trust of* ${n_{i}}$ *related to* ${n_{j}}$ (${T_{ij}^{indirect}}$)**:** $T_{ij}^{indirect}$ characterizes the trust amount obtained from the recommended nodes, which are the common and reliable neighbors between $n_{i}$ and $n_{j}$. The recommended nodes should be picked out from reliable nodes whose trust amount is more than $T_{threshold}$. We assume that there is a set called *R*, which includes *p* recommended nodes between $n_{i}$ and $n_{j}$ so that $R=\left\{ n_{Recommender}^{1},n_{Recommender}^{2},...,n_{Recommender}^{p}\right\}$. In this case, $T_{ij}^{indirect}$ is obtained using Eq. (11).
  11 $$\begin{aligned} T_{ij}^{indirect}=\frac{1}{p}\sum \limits _{x\in R}^{p}{\left( T_{ix}^{direct}\cdot T_{xj}^{direct}\right) } \end{aligned}$$
  where $T_{ix}^{direct}$ and $T_{xj}^{direct}$ express the direct trust of $n_{i}$ to $n_{x}$ and the direct trust of $n_{x}$ to $n_{j}$, respectively. Also, $n_{x}$ indicates a recommended node. The MF of $T_{ij}^{indirect}$ is displayed in Fig. 4. $T_{ij}^{indirect}$ contains three modes, including low, medium, and high.

#### Fuzzy output

**Figure 5:**
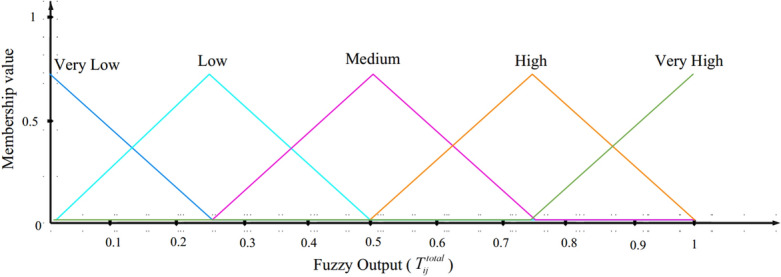
MF of $$T_{ij}^{total}$$.

The output of this fuzzy trust framework illustrates the total trust ($T_{ij}^{total}$), which includes five modes (very low, low, medium, high, and, very high). See the MF of $T_{ij}^{total}$ in Fig. 5.

#### Rule base

The proposed trust framework defines the rules presented in Table 2. For example, Rule 1 is stated below.

*Rule 1:* **IF** $T_{ij}^{direct}$ is *low* **AND** $T_{ij}^{indirect}$ *low* **THEN** $T_{ij}^{total}$ is *very low*.

**Table 2:** Rule base.

Number	$\mathrm {T_{ij}^{direct}}$	$\mathrm {T_{ij}^{indirect}}$	$\mathrm {T_{ij}^{total}}$
1	Low	Low	Very low
2	Low	Medium	Low
3	Low	High	Medium
4	Medium	Low	Low
5	Medium	Medium	Medium
6	Medium	High	High
7	High	Low	Medium
8	High	Medium	High
9	High	High	Very high


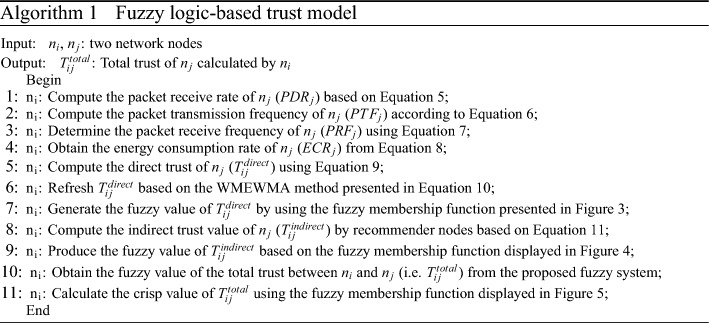


### Firefly algorithm-based clustering framework

Here, the firefly algorithm-based clustering framework is stated in FSRF. This framework will be executed by the base station. Algorithm 2 describes the clustering framework in FSRF. This framework comprises two steps:

- Clustering
- Cluster maintenance

#### Clustering

Each IoT node, like ${n_{i}}$, transfers a guide message to BS. The message is named a beacon, which contains the trust amount, location, remaining energy, hops to BS, centrality degree, and communication radius. Next, BS assesses the trust of ${n_{i}}$ to differentiate the trusted nodes from untrusted nodes. Note that the trusted nodes have high trust (more than ${T_{threshold}}$). In FSRF, only trusted nodes can be cluster heads. Then, the BS starts the FA-based CH selection algorithm. In this algorithm, each firefly plays the role of an IoT node (${n_{i}}$), and the value of this firefly states the chance of ${n_{i}}$ to behave as CH between neighboring nodes. In the first step, the value of each firefly is a random number. Here, each ${n_{i}}$ expresses a firefly, which may be a CH. It presents a response to the CH selection problem. The primary attractiveness of the fireflies is displayed as ${{\beta }_{0}}$ determined by the RAND function. In this CH selection framework, BS has an important responsibility, which must employ the FA algorithm to decide on the best CHs in the network. Thereafter, the base station will assess the fitness amount of each response (firefly) based on an objective function. This function is formulated in accordance with five parameters, namely the trust amount, remaining energy, hops to BS, communication radius, and the average distance to the neighboring nodes.

- *Trust amount *(${{T^{total}}}$): The purpose of this parameter in the objective function is to select secure nodes as a CH because cluster heads have important tasks including the submission of data packets received from CMs, participation in the routing process between CHs, and the transmission of data packets of other CHs to the base station. Therefore, if an insecure node is selected as a CH, it can damage the normal performance of the network. The BS extracts ${T^{total}}$ from the guide message received from ${n_{i}}$. “Fuzzy trust framework” section explains how to calculate ${T^{total}}$ in detail. If ${n_{i}}$ utilizes a higher trust amount, it has a greater chance to act as a CH because it is safer and can send the data to the network reliably. ${T^{total}}$ will be normalized by Eq. (12).
  12 $$\begin{aligned} T_{norm}^{total}=\frac{{T^{total}}-{T_{thershold}}}{{T_{\max }}-{T_{thershold}}} \end{aligned}$$
  So that ${T_{thershold}}$ and ${T_{\max }}$ are the minimum acceptable trust determined for CHs and the maximum trust amount in the network, respectively.
- *Remaining energy * (${{E_{r}}}$)**:** The purpose of this parameter in the objective function is that energy consumption in the network is evenly distributed between the IoT nodes to increase network longevity. In this regard, high-energy nodes have a responsibility to play the role of cluster heads because CHs have more responsibilities than normal nodes and consume more energy. If low-energy nodes play the role of CH, their energy will be ended quickly. In this case, finding the new CH is also accompanied by a lot of cost, time, and communication overhead. The BS extracts ${E_{r}}$ from the guide message obtained from ${n_{i}}$. Energy is very important when deciding on CHs because IoT nodes suffer from energy restrictions on the network. Additionally, network nodes have different amounts of energy. Consequently, if ${n_{i}}$ has more energy than other nodes on the network, ${n_{i}}$ gets a higher chance to act as CH. ${E_{r}}$ is normalized according to Eq. (13).
  13 $$\begin{aligned} E_{r}^{norm}=\frac{{E_{r}}-{E_{\min }}}{{E_{\max }}-{E_{\min }}} \end{aligned}$$
  As, ${E_{r}}$, ${E_{\min }}$, and ${E_{\max }}$ are the remaining energy of ${n_{i}}$, the lowest energy level, and the maximum energy level of the network nodes, respectively.
- *Hops to BS *(${{H_{c}}}$)*:* ${H_{c}}$ is very important in the decision-making process for CHs because if CHs have fewer hops to the BS, the data packets reach the BS faster and experience less delay. ${H_{c}}$ is normalized using Eq. (14).
  14 $$\begin{aligned} H_{c}^{norm}=\frac{{H_{c}}-{H_{\min }}}{{H_{\max }}-{H_{\min }}} \end{aligned}$$
  So that ${H_{c}}$ indicates the hop count from ${n_{i}}$ to the BS, ${H_{\min }}$ represents the minimum hops to the BS, where ${H_{\min }}=1$, and ${H_{\max }}$ expresses the maximum hops to the BS, which is dependent on the number of nodes (i.e. *N*) so that ${H_{\max }}=N-1$.
- *Communication radius * (${{R_{com}}}$): IoT nodes are heterogeneous and ${R_{com}}$ is different. In FSRF, this subject is intended in the CH selection framework, so that ${n_{i}}$ with a large ${R_{com}}$ gets a greater chance to act as CH because ${n_{i}}$ covers a wider range. ${R_{com}}$ is normalized based on Eq. (15).
  15 $$\begin{aligned} R_{com}^{norm}=\frac{{R_{com}}-{R_{\min }}}{{R_{\max }}-{R_{\min }}} \end{aligned}$$
  So that ${R_{com}}$, ${R_{\min }}$, and ${R_{\max }}$ are the communication radius of ${n_{i}}$, the least radius, and the highest radius, respectively.
- *Average distance to neighboring nodes* (${{D_{avg}}}$): ${D_{avg}}$ shows the centrality of ${n_{i}}$ in the cluster. When ${n_{i}}$ is close to the cluster center, ${D_{avg}}$ is low. In this case, if a node close to the cluster center is selected as a CH, this increases energy efficiency in the network because the average distance between this CH and its CMs is reduced and the CH needs less energy to receive the data packets from CMs. This parameter will be calculated using Eq. (16).
  16 $$\begin{aligned} {D_{avg}}=\frac{1}{{T_{i}}}\sum \limits _{u\in Nei}^{{T_{i}}}{d\left( {n_{i}},u \right) } \end{aligned}$$
  Where ${T_{i}}$ indicates the number of neighbors of ${n_{i}}$. Furthermore, $d\left( {n_{i}},u \right)$ is the distance from ${n_{i}}$ to its neighbor (*u*). $d\left( {n_{i}},u \right)$ is obtained through Eq. (17).
  17 $$\begin{aligned} d\left( {n_{i}},u \right) =\sqrt{{{\left( {x_{i}}-{x_{u}} \right) }^{2}}+{{\left( {y_{i}}-{y_{u}}\right) }^{2}}} \end{aligned}$$
  Where, $\left( {x_{i}},{y_{i}}\right)$ and $\left( {x_{u}},{y_{u}}\right)$ are the positions of ${n_{i}}$ and *u*, respectively. Finally, ${D_{avg}}$ is normalized using Eq. (18).
  18 $$\begin{aligned} D_{avg}^{norm}=\frac{{D_{avg}}-{D_{\min }}}{{D_{\max }}-{D_{\min }}} \end{aligned}$$
  Where, ${D_{\min }}$ and ${D_{\max }}$ are the shortest and longest distances of neighboring nodes on the network, respectively.

As a result, the objective function is calculated in accordance with Eq. (19).

19 $$\begin{aligned} F=\frac{{{\lambda }_{1}}T_{norm}^{total}+{{\lambda }_{2}}E_{r}^{norm}+{{\lambda }_{3}}R_{com}^{norm}}{{{\alpha }_{1}}H_{c}^{norm}+{{\alpha }_{2}}D_{avg}^{norm}} \end{aligned}$$

So that ${{\lambda }_{1}}$, ${{\lambda }_{2}}$, ${{\lambda }_{3}}$, ${{\alpha }_{1}}$, and ${{\alpha }_{2}}$ represent the weight coefficients where, $\sum \limits _{i=1}^{3}{{{\lambda }_{i}}}=1$ and $\sum \limits _{i=1}^{2}{{{\alpha }_{i}}}=1$. In Eq. (19), $T_{norm}^{total}$, $E_{r}^{norm}$, $R_{com}^{norm}$, $H_{c}^{norm}$, and $D_{avg}^{norm}$ are normalized in $\left[ 0,1 \right]$ to have the same effect on the objective function.

After determining the chance of each firefly (IoT node), the position of these fireflies is renewed using the firefly algorithm. In the FA-based CH selection framework, the algorithm is repeated 300 times (stop condition). Upon the FA-based clustering framework has ended, the BS picks out the best firefly with the highest fitness amount as a CH. Then, BS informs IoT nodes of their roles. Next, CHs prepare a notification message to inform their neighboring nodes of their roles on the network. This notification message includes the coordinates of CHs. When neighboring nodes get these notification messages, CHs must recognize their members. In the first mode, when an ordinary node gets one or more notification messages from different CHs, it sends a membership request to the nearest CH. In the second mode, when an ordinary node with a trust amount more than ${T_{threshold}}$ does not get any notification message from CHs, it acts as a CH and broadcasts a notification message to recognize. Now, there are two types of nodes in the network: CHs and CMs. The cluster member nodes are only associated with their CH and transfer their data to it. However, the CHs communicate directly with CMs and their neighboring CH.

#### Cluster maintenance

The purpose of the cluster maintenance process is to adjust the role of each node through the periodic exchange of guide messages so that the connections can be stable on the network. All IoT nodes participate in the cluster maintenance process. This process includes the following steps:

- *Connect to the cluster:* When a new IoT node is connected to the network, it broadcasts a membership request. A CH node that receives this request faster than other CHs, responds to it and accepts the membership of this new node in its cluster.
- *Leave the cluster:* Each CM examines its connection with its CH through the periodic exchange of guide messages. If this link is invalid, CM has been removed from membership in the cluster, and again, the CM broadcasts a membership request on the network to connect to the nearest CH.
- *Cluster membership checking:* Each CH examines its communication with its CMs through the periodic exchange of guide messages with its CMs. If the communication links are invalid, the CH should cancel the membership of the desired CM in its cluster.
- *Re-clustering:* The status of IoT nodes changes depending on their energy levels or their trust over time. As a result, it is necessary for the BS to constantly monitor their status by periodic guide messages received from IoT nodes. If CHs lose their energy or trust amount, it transfers a cluster update message to its CMs and re-runs the FA-based selection framework in “Firefly algorithm-based clustering framework” section. In this case, the IoT nodes are waiting for the FA-based clustering operation to be implemented by the base station to choose a new CH.

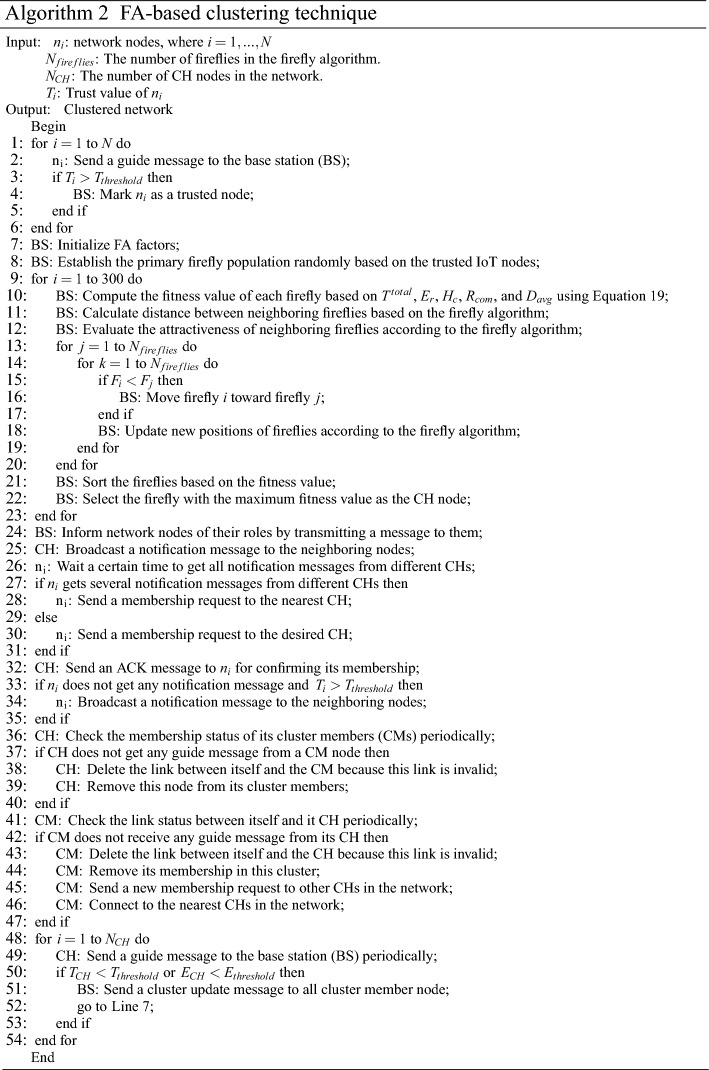


### Inter-cluster routing framework

In FSRF, the inter-cluster routing framework consists of two main steps: discovering paths between CHs and maintaining these paths. Algorithm 3 presents the pseudo-code related to the inter-cluster routing framework.

#### Discovering paths between CHs

**Figure 6:**
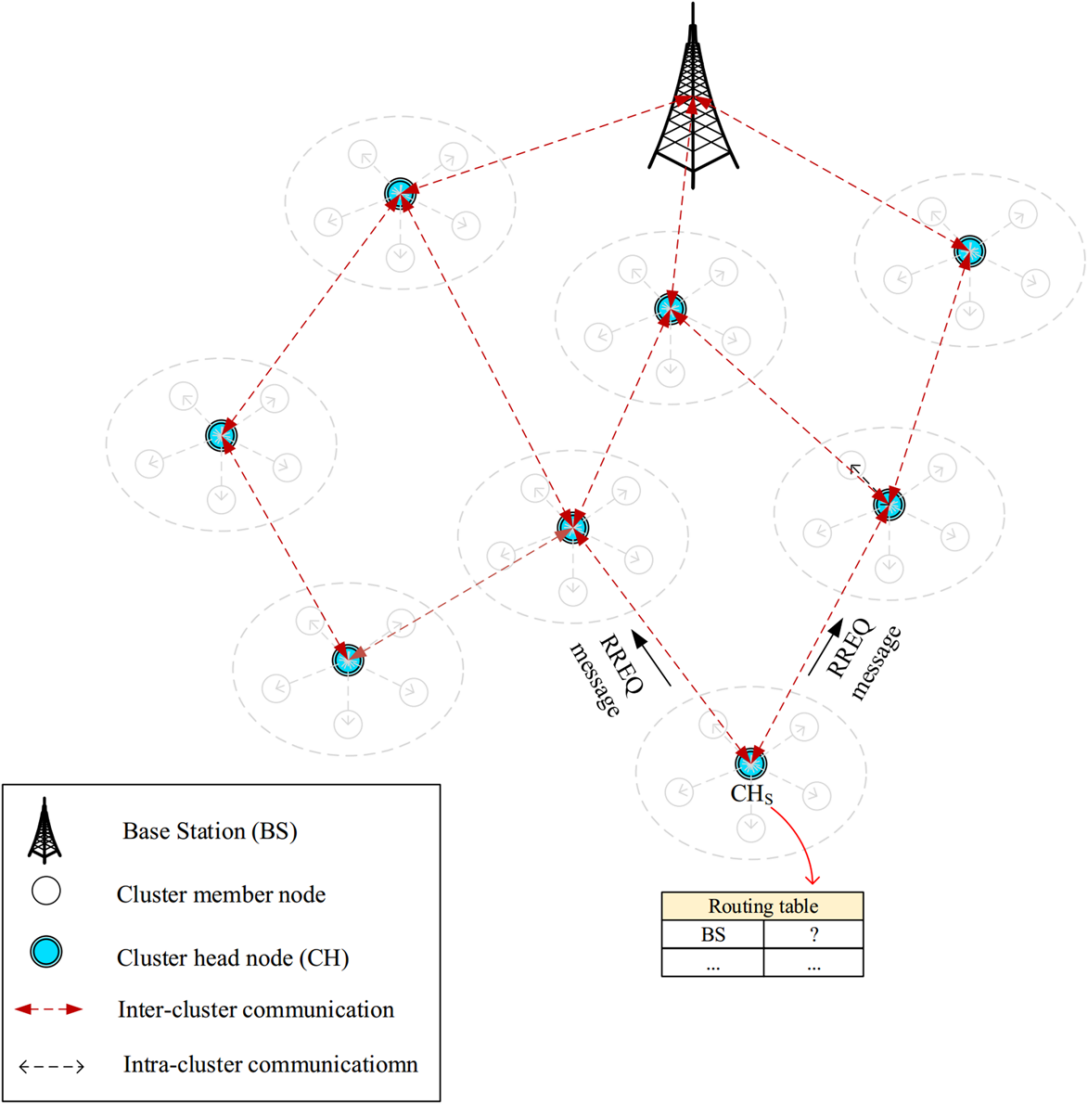
Spreading RREQ message.

FSRF executes an on-demand technique for discovering paths. When a cluster head, like $CH_{S}$, is looking for a path to the BS. In the first stage, its routing table is searched, and $CH_{S}$ examines whether it can find a connected path to the BS. If yes, $CH_{S}$ connects to the BS through this path to transmit data packets. Otherwise, $CH_{S}$ begins a path search operation to find a safe and energy-efficient path. In this operation, $CH_{S}$ creates a route request (RREQ) and spreads it to its one-hop neighbors. View Fig. 6.

**Figure 7:**
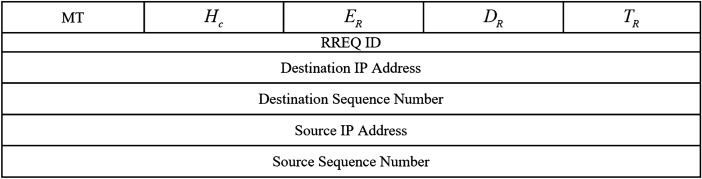
RREQ message format.

See the format of RREQ in Fig. 7. The fields of this message are explained below:

- *Message type (MT):* If $MT=1$, this control message indicates a RREQ
- ${\textbf{H}_{c}}$ **:** It counts hops in the relevant path. $CH_{S}$ adjusts the initial amount of $H_{c}$ to zero and then, each intermediate node adds one unit to $H_{c}$. In RREQ, $H_{c}$ helps prevent the formation of routing loops in the created paths.
- *RREQ ID:* Each RREQ is marked with a special ID. This ID and the $CH_{S}$ address are used for checking RREQs and finding repeated RREQs.
- ${{\textbf{E}_{R}}}$: It determines how much energy is consumed to send data from $CH_{S}$ to the desired node through the relevant path. In general, $E_{R}$ is computed using Eq. (20).
  20 $$\begin{aligned} {E_{R}}={E_{consumed}}\left( Source \right) +\sum \limits _{CH_{intermediate}\in Route_{k}}^{Destination-1}{{E_{consumed}}\left( CH_{intermediate}\right) }+{E_{consumed}}\left( Destination \right) \end{aligned}$$
  so that ${E_{consumed}}\left( Source \right)$ is the energy used in $CH_{S}$ for transferring RREQ, ${E_{consumed}}\left( CH_{intermediate}\right)$ is the required energy of intermediate nodes to get and forward RREQ, ${E_{consumed}}\left( Destination\right)$ is the required energy of the desired node for getting RREQ. According to the energy model stated in “Energy model” section, Eq. (20) is rewritten as Eq. (21).
  21 $$\begin{aligned} {E_{R}}=E_{tx}^{Source}+\sum \limits _{CH_{i}\in Route_{k}}^{Destination-1}{\left( E_{tx}^{CH_{i}}+E_{rx}^{CH_{i}}\right) }+E_{rx}^{Destination} \end{aligned}$$
  where ${E_{tx}}$ and ${E_{rx}}$, which are respectively obtained from Eqs. (2) and (4), are the required energy for transferring and getting RREQs.
- ${{\textbf{D}_{R}}}$**:** This field stores the total delay taken from $CH_{S}$ to the desired node (i.e. base station). The initial amount of ${D_{R}}$ is zero. In the next hops, the amount of ${D_{R}}$ is dependent on ${D_{Propagation}}$, ${D_{Queuing}}$, ${D_{Computing}}$, and ${D_{Transmission}}$. In this regard, the value of ${D_{R}}$ is refreshed using Eq. (22).
  22 $$\begin{aligned} {D_{R}}=\sum \limits _{i=Source}^{Destination}{\left( {D_{Propagation}}\left( CH_{i} \right) +{D_{Queuing}}\left( CH_{i}\right) +{D_{Computing}}\left( CH_{i}\right) +{D_{Transmission}}\left( CH_{i}\right) \right) } \end{aligned}$$
  So, *Source* and *Destination* are the source and destination nodes, respectively. Note that ${D_{Propagation}}$ is the time taken for transferring data through the wireless link between two intermediate nodes. There is a direct relationship between ${D_{Propagation}}$ and the distance between the two nodes. ${D_{Propagation}}$ is derived from Eq. (23).
  23 $$\begin{aligned} {D_{Propagation}}=\frac{Dist\left( CH_{i},CH_{j}\right) }{{v_{media}}} \end{aligned}$$
  So that ${v_{media}}$ is the light speed (i.e. $3\times {10^{8}}$) and $Dist\left( CH_{i},CH_{j}\right)$, which is calculated using Eq. (24), indicates the distance from $CH_{i}$ to $CH_{j}$.
  24 $$\begin{aligned} Dist\left( CH_{i},CH_{j}\right) =\sqrt{{{\left( {x_{i}}-{x_{j}}\right) }^{2}}+{{\left( {y_{i}}-{y_{j}} \right) }^{2}}} \end{aligned}$$
  So that $\left( {x_{i}},{y_{i}}\right)$ and $\left( {x_{u}},{y_{u}}\right)$ express the positions of $CH_{i}$ and $CH_{j}$, respectively. In addition, ${D_{Queuing}}$ states a time interval when RREQs have to wait in the buffer queue to send. ${D_{Computing}}$ represents the time required to process RREQ in the relevant node. In addition, ${D_{Transmission}}$ indicates the time taken for transferring RREQ to the next CH. It is obtained according to Eq. (25).
  25 $$\begin{aligned} {D_{Transmission}}=\frac{ms{g_{size}}}{br} \end{aligned}$$
  So that *br* and $msg_{size}$ demonstrate the transfer rate and the size of RREQ, respectively.
- ${{\textbf{T}_{R}}}$**:** It determines whether a path is reliable. Initially, $CH_{S}$ adjusts the amount of ${T_{R}}$ to its trust amount. Then, ${T_{R}}$ will be updated in accordance with Eq. (26) in each hop. The amount of ${T_{R}}$ is considered the lowest trust amount of CHs in a routing path. ${T_{R}}$ helps $CH_{S}$ to prevent the selection of insecure paths.
  26 $$\begin{aligned} {T_{R}}=\underset{CH_{i},CH_{j}\in Route_{k}}{\mathop {\min }}\,\left( T_{ij}^{total}\right) \end{aligned}$$
  where $CH_{i}$ and $CH_{j}$ show the pervious-hop and next-hop intermediate CHs in the present path (i.e. $Route_{k}$), respectively. Also, $T_{ij}^{total}$ is the trust amount of $CH_{i}$ to $CH_{j}$ explained in “Fuzzy trust framework” section.
- *Source IP address (SIA):* It specifies the address of $CH_{S}$.
- *Destination IP address (DIA):* It specifies the address of the BS.
- *Source sequence number (SSN):* It helps intermediate nodes to ensure that the information about the reversed path to $CH_{S}$ is new.
- *Destination sequence number (DSN):* Before choosing a path by $CH_{S}$, DSN guarantees that the path is new.

**Figure 8:**
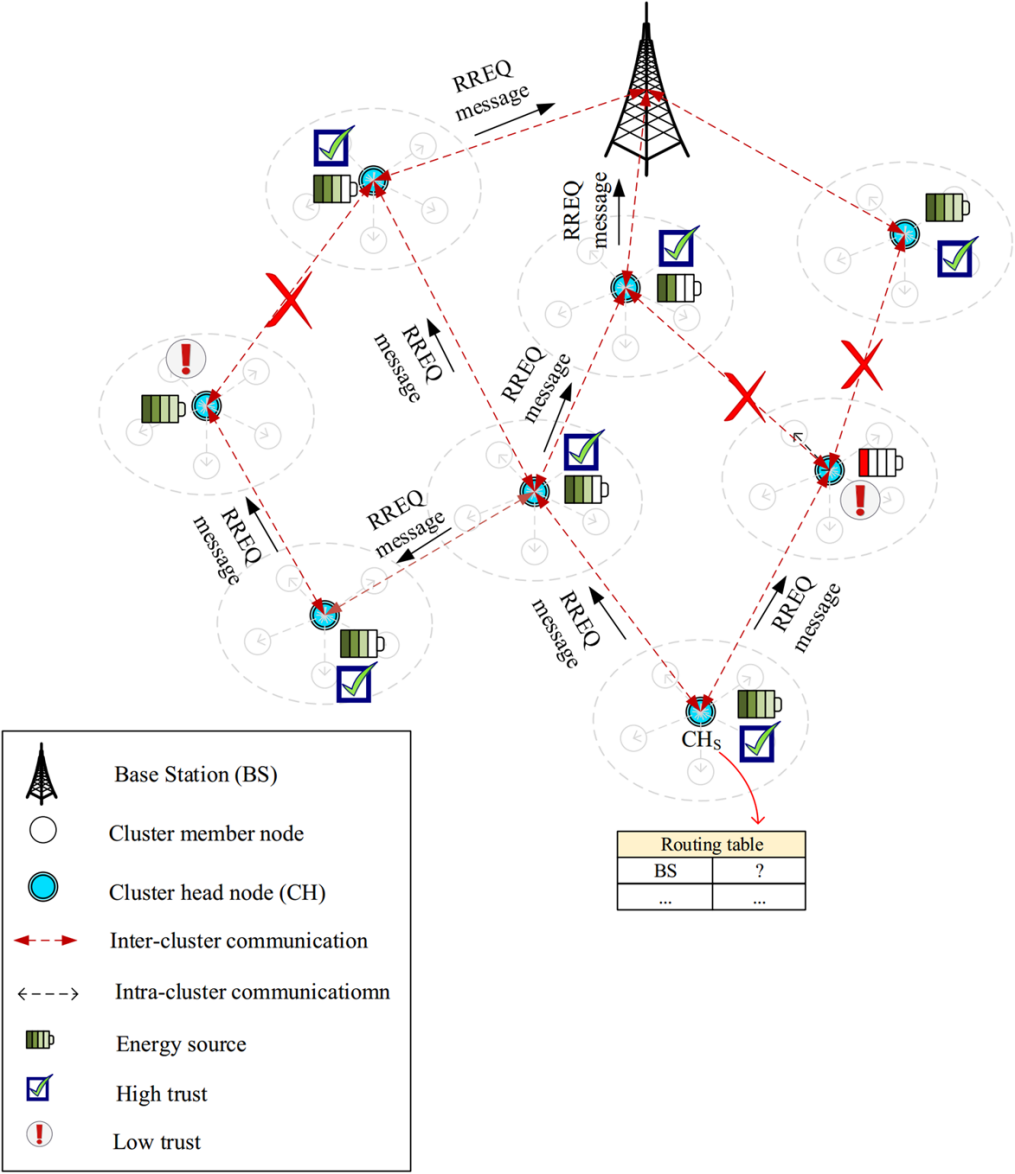
Path search process.

After an intermediate CH gets RREQ, it first examines its ID and ensures that the RREQ is not old. Next, the intermediate node compares its remaining energy (${E_{r}}$) and its trust amount (${T^{total}}$) with ${E_{threshold}}$ and ${T_{threshold}}$, respectively. If ${E_{r}}>{E_{threshold}}$ and ${T^{total}}>{T_{threshold}}$, the CH is allowed to rebroadcast the RREQ. Otherwise, this CH should delete the RREQ. This strategy will improve the performance of the routing method in terms of energy efficiency and security. Once the RREQ reaches the base station, the RREQ broadcast process ends. See this process in Fig. 8.

**Figure 9:**
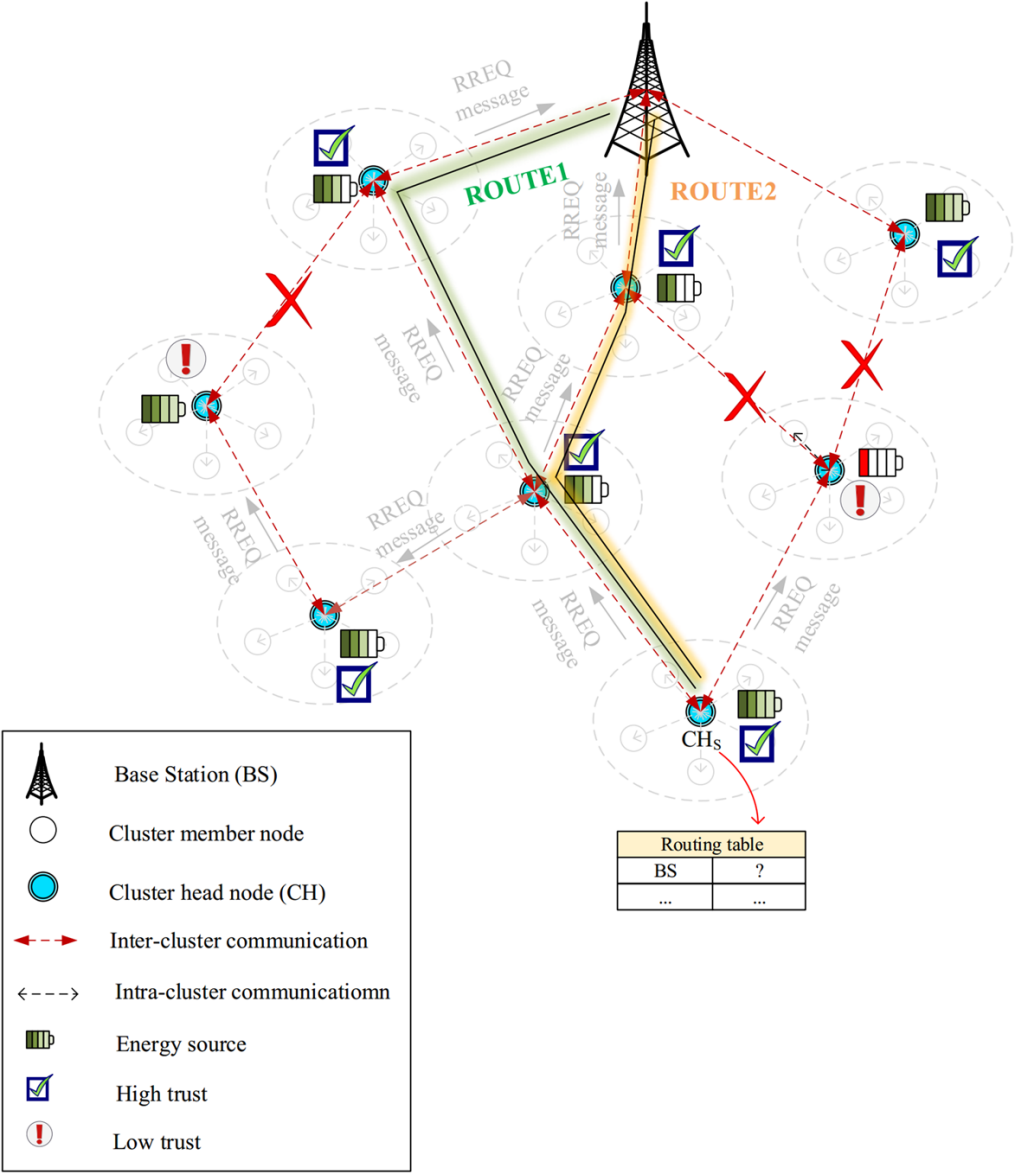
Paths found between $CH_{S}$ and the BS.

Now, the base station must choose one path from the formed paths between $CH_{S}$ and itself. For example, see ROUTE1 and ROUTE2 in Fig. 9. In the route selection operation, the base station utilizes the information recorded in RREQs to calculate the score of each path based on Eq. (27).

27 $$\begin{aligned} {S_{R}}=\frac{{T_{R}}}{{E_{R}}+{D_{R}}+{H_{c}}} \end{aligned}$$

So that ${T_{R}}$, ${E_{R}}$, ${D_{R}}$, and ${H_{c}}$ are the path trust (Eq. 27), the energy consumed in the path (Eq. 21), the delay taken in the path (Eq. 22), and hops counted in the path, respectively.

**Figure 10:**
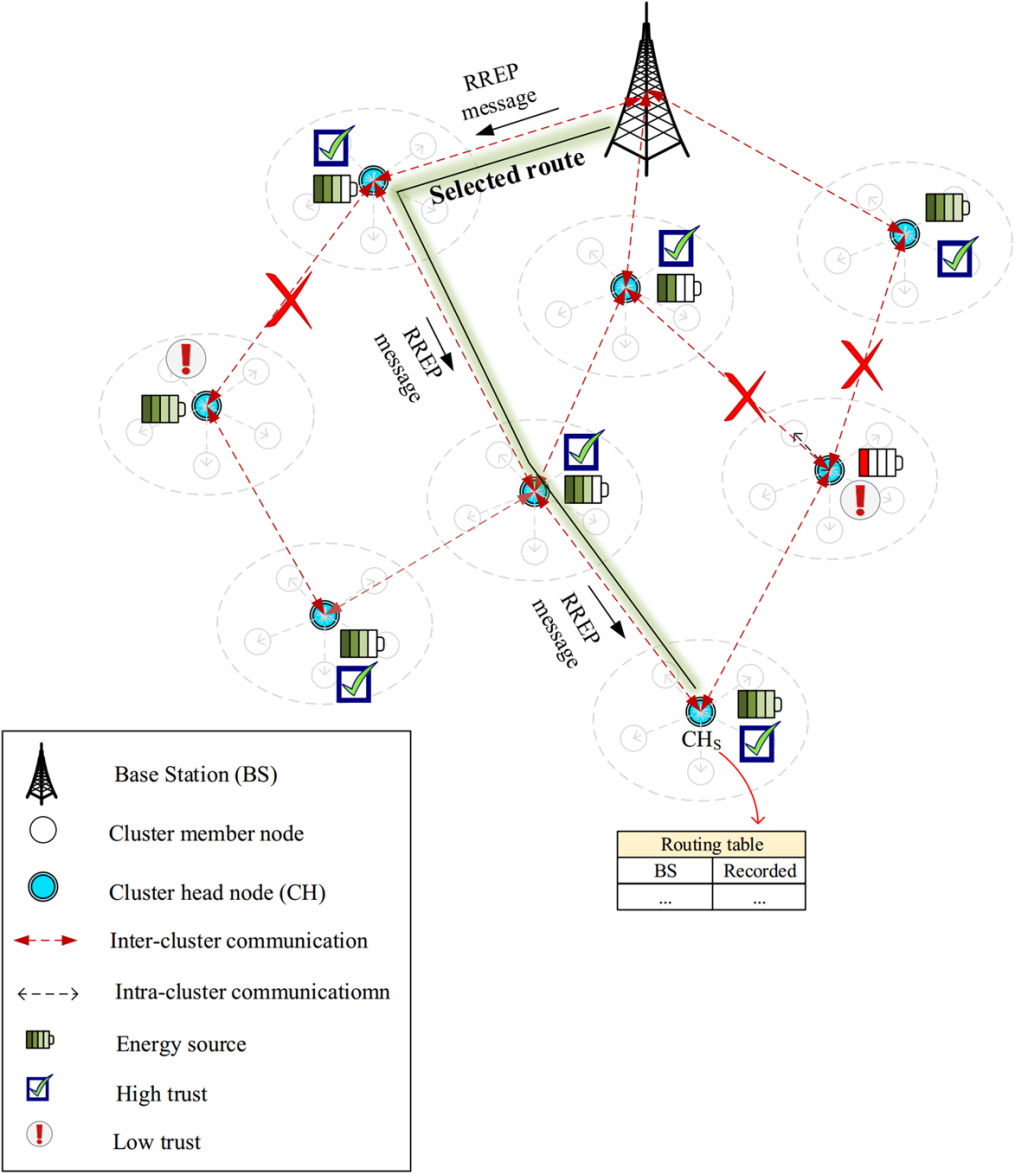
Sending the RREP message.

Then, the BS chooses the high-score path to transfer the data between $CH_{S}$ and itself. Finally, the base station builds a route reply (RREP) message and transmits it for $CH_{S}$ through the determined path. After receiving RREP, $CH_{S}$ records the path in its table and uses it to transfer data to BS. See this process in Fig. 10.

#### Maintaining the formed paths

The path maintenance process is carried out to determine whether the formed path is cut (i.e. the route discovery process must be started again) or the formed path is connected. CHs regularly examine the connection status of the paths available in their routing table. For this reason, $CH_{S}$ carries out the periodic transmission of a route validation message through the existing path. If the BS gets the message, it will confirm the connection of the path and transfers an acknowledgment (ACK) to $CH_{S}$. Otherwise, if $CH_{S}$ does not receive any confirmation message from the BS for a certain period of time, $CH_{S}$ is aware of the disconnection of the existing path and will begin the path search operation again in accordance with “Inter-cluster routing framework” section.
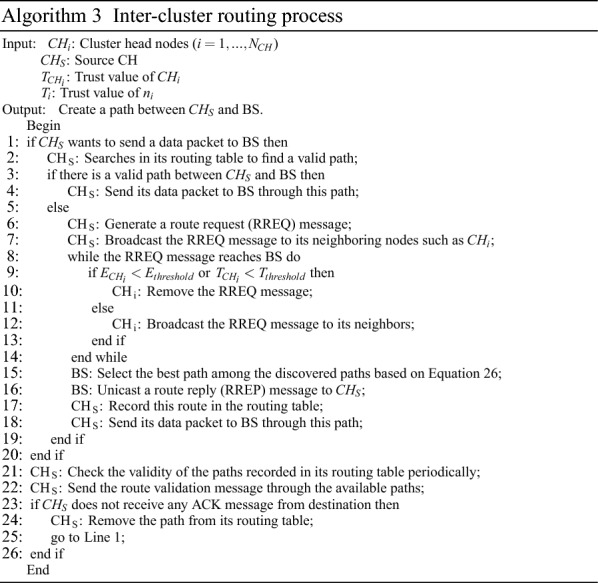


## Simulation and result evaluation

To accurately analyze the performance of FSRF, it must be carefully simulated and evaluated in different scenarios. For reaching this goal, the simulation operation is run in Network Simulator 2 (NS2) in accordance with the parameters listed in Table 3. According to the information recorded in this table, it can be found that the dimensions of the simulation environment are $100\times 100\,{{\text{m}}^{2}}$. In this process, 100 IoT nodes are randomly deployed in the simulation environment. These IoT nodes are immobile. They have heterogeneous energy sources, so there are 50 nodes with 2*J* energy, 35 nodes with 4 J energy, 12 nodes with 5 J energy, and 3 nodes with 6 J energy in the network. The transfer radius of these nodes is 20 meters. In FSRF, it is assumed that 10% of the nodes are hostile that are randomly selected from the network nodes with different energy levels. Each round includes 100 data transfer operations and each packet is 500 bytes. In this section, five test criteria are considered:

- *Criterion 1) Trust status:* This criterion evaluates the trust amount of the network nodes, whether hostile or normal, after various rounds and the exchange of information between the network nodes.
- *Criterion 2) Network longevity:* This criterion is used to analyze the lifetime of the network by counting the number of dead nodes in the network after various rounds.
- *Criterion 3) Energy level evaluation:* This criterion is used to measure the energy stored in the nodes after various rounds.
- *Criterion 4) Energy balance:* This criterion is used to evaluate whether the consumed energy is distributed between the network nodes evenly. To achieve this goal, the standard deviation of the energy consumed in nodes ($SD_{Energy}$) is calculated. If $SD_{Energy}$ is close to zero, it confirms the balanced consumed energy between network nodes. However, if $SD_{Energy}$ is close to one, it shows an imbalance energy consumption in the network.
- *Criterion 5) Packet delivery rate:* This criterion is for measuring the total number of data packets received at the destination compared to all packets sent from $CH_{S}$.

We compare FSRF to EEMSR and E-BEENISH. The selection of these two methods has several reasons:

- FSRF, EEMSR, and E-BEENISH are hierarchical and use clustering techniques to enhance energy efficiency in the network.
- EEMSR and FSRF have used metaheuristic algorithms to rise the performance of the IoT network so that EEMSR employs a genetic algorithm to correct the routing framework and FSRF employs the firefly algorithm to enhance the clustering process.
- EEMSR and FSRF have provided powerful trust mechanisms to protect the network nodes. However, E-BEENISH has not considered any security mechanism.

**Table 3:** Simulation settings.

Parameter	Value
Simulation tool	NS2
The dimensions of network	$100\times 100\,{\text{m}^{2}}$
BS position	$\left( 50,100 \right)$
Total number of nodes	100
The number of nodes with 2 J energy	50
The number of nodes with 4 J energy	35
The number of nodes with 5 J energy	12
The number of nodes with 6 J energy	3
Transfer radius	$20\,\,\text{m}$
Primary trust amount	0.5
Packet size	500 Bytes
The number of the data transfer operation in each round	100
${E_{elec}}$	$50\,{\text{nJ}}/{\text{bit}}$
${{\epsilon }_{fs}}$	10 pJ/bit/m^2^
${{\epsilon }_{mp}}$	0.0013 pJ/bit/m^4^
The primary population of fireflies	80
The number of iterations in FA	$300\,\,times$

### Trust amount

**Figure 11:**
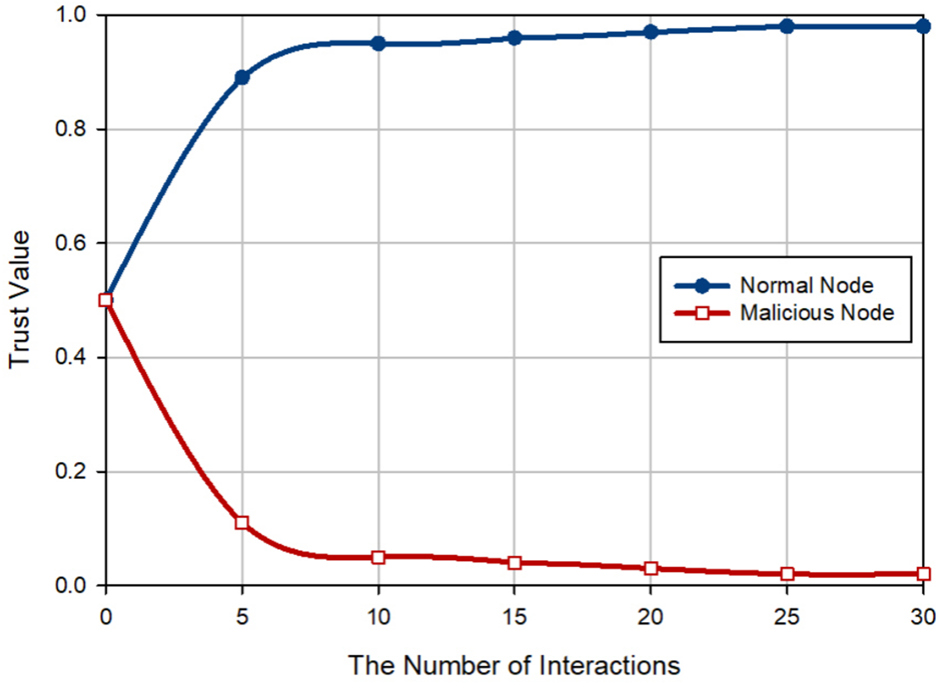
The performance measurement of FSRF based the trust evaluation.

The first criterion for examining the performance of FSRF is to evaluate the trust amount of the network nodes. The results of this evaluation are displayed in Fig. 11. Note that there are two hypotheses in the performance measurement process: (1) The invading nodes are present in the network (i.e. 10% of the total network nodes) and (2) the initial trust of the nodes is adjusted to 0.5. Figure 11 shows when launching the network, it is difficult to distinguish the hostile nodes from the honest nodes because the exchange of information between the nodes is low and their trust is not well known. After increasing the exchange between the nodes, the fuzzy trust system designed in FSRF can help the nodes to be accurately aware of the trust status of themselves and their neighboring nodes. This increases the trust of the honest nodes to one and reduces the trust of hostile nodes to zero. In this case, FSRF can well separate hostile nodes from honest nodes.

### Network longevity

**Figure 12:**
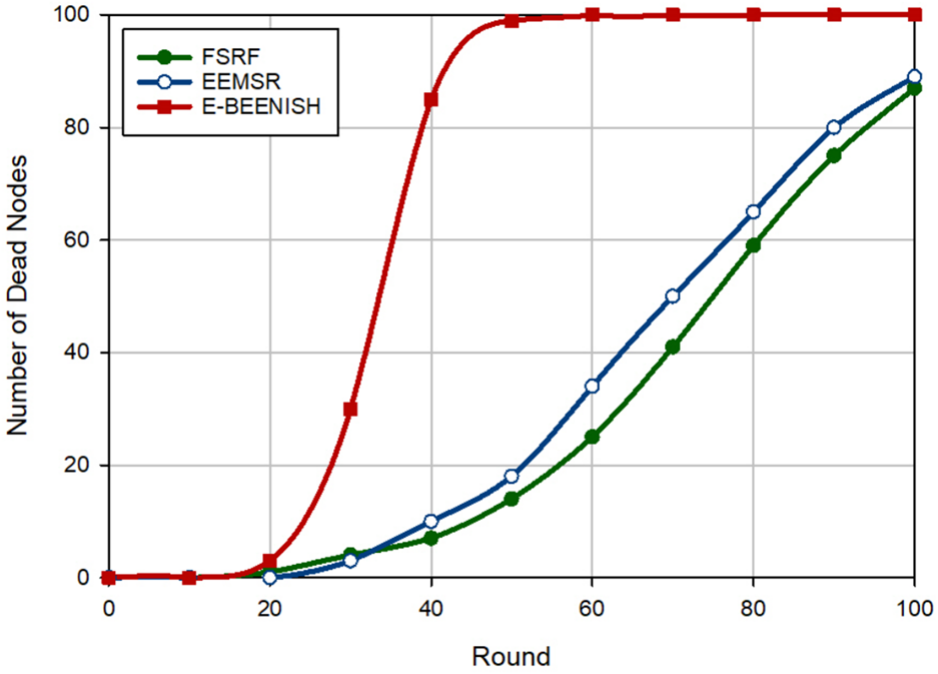
Evaluation of network longevity.

**Figure 13:**
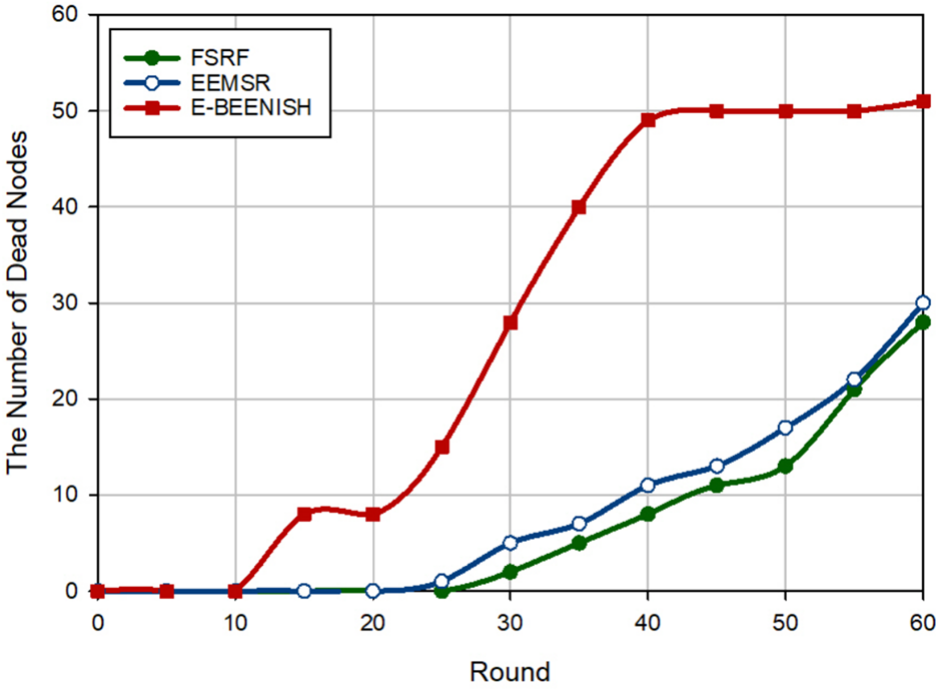
Evaluation of network longevity by changing the location of the BS to the corner of the network.

**Figure 14:**
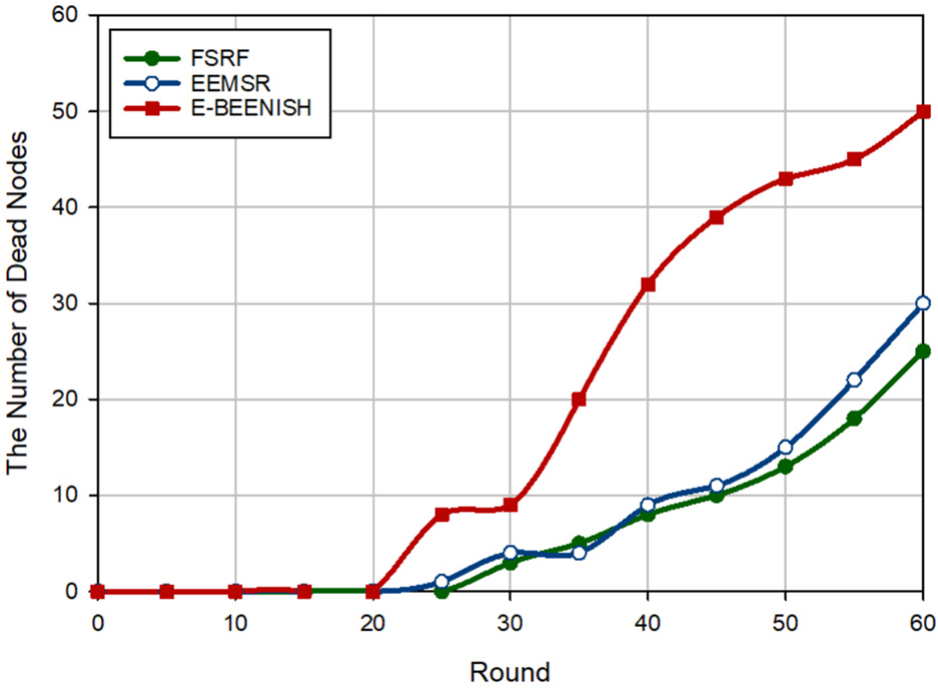
Evaluation of network longevity by changing the location of the BS to the center of the network.

The second criterion for examining the performance of FSRF is network longevity, which is obtained by counting the number of dead nodes at each round. In Fig. 12, FSRF has achieved the best network longevity compared to EEMSR (approximately 10.34%) and E-BEENISH (approximately 56.35%). Note that the performance of EEMSR and FSRF is very close to each other in terms of network longevity. If network longevity is defined based on the first node die (FND), EEMSR is superior to FSRF. However, if network longevity is defined based on half of the nodes die (HND) or the last node die (LND), the performance of FSRF is better than that of EEMSR and E-BEENISH. Now, this experiment is repeated by changing the location of the BS to analyze its effect on the routing schemes. In Fig. 13, the BS position is in the corner of the network, i.e. $\left( 0,0 \right)$. In this case, counting the number of dead nodes at each round indicates the improvement of this criterion by FSRF in comparison with EEMSR (16.93%) and E-BEENISH (74.79%). In Fig. 14, the BS is placed at the center of the network, i.e. $\left( 50,50 \right)$. In this figure, FSRF has succeeded in improving this criterion compared to EEMSR (14.50%) and E-BEENISH (66.65%). In these two experiments (Figs. 13 and 14), FSRF is superior to EEMSR in terms of FND. Additionally, the two experiments prove that changing the position of BS in FSRF and EEMSR cannot have a great impact on their performance. As a result, they are adaptable. However, this change in position has affected the performance of E-BEENISH. The better performance of FSRF in terms of network longevity is rooted in the attention to energy and security at the same time because FSRF only allows the trusted nodes with high energy to broadcast RREQ. Furthermore, FSRF has taken into account the amount of energy consumed in the discovered routes in the route selection process, but EEMSR does not pay attention to this parameter in the routing process. In addition, in the clustering process, the high-energy nodes are selected as CHs. E-BEENISH has not paid attention to the security of the network, so invaders can reduce network longevity by impacting the network performance. In E-BEENISH, each CH must send data packets to the BS in a one-hop way, which requires a lot of energy. However, EEMSR and FSRF have used intermediate nodes to send data to the base station. This improves energy balance in the network and thus extends network longevity.

### Energy

**Figure 15:**
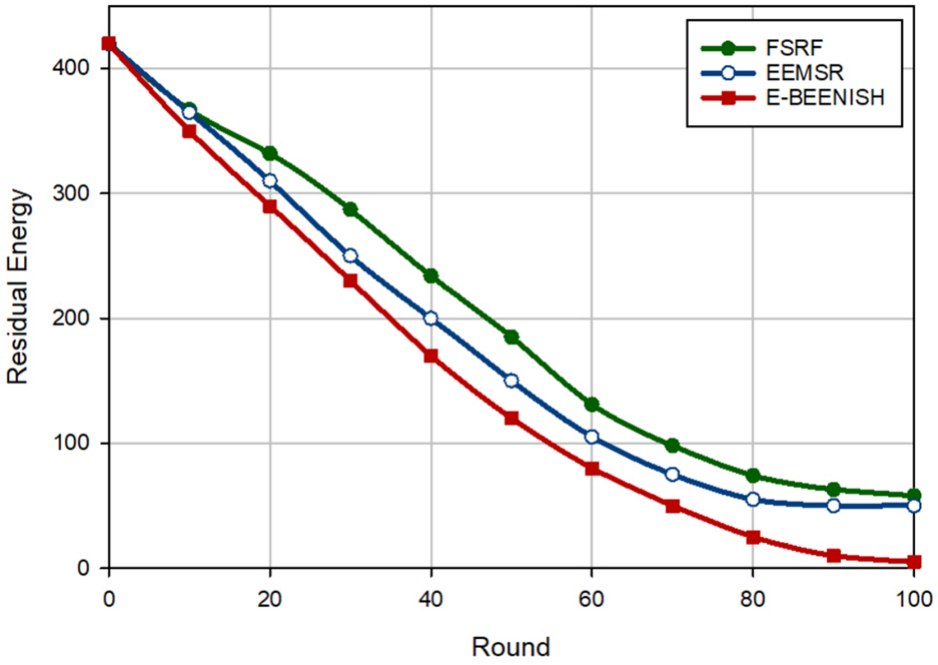
Evaluation of energy stored in nodes.

The third criterion for examining the performance of FSRF is to evaluate the amount of energy stored in the network nodes. See Fig. 15. FSRF improves the energy stored in the nodes by 10.79% and 28.51%, compared to EEMSR and E-BEENISH, respectively. Given this figure, it can be said that FSRF and EEMSR have a good performance in terms of energy stored in the nodes. However, the E-BEENISH has a weaker performance in this criterion because E-BEENISH does not pay attention to the energy of the nodes in the CH selection process, as well as the direct transmission of data from each CH to BS has reduced the energy stored in the nodes. Lack of attention to network security can also reduce the energy stored in nodes due to the selection of untrusted CHs, the need to re-transfer data, and high packet loss. However, EEMSR and FSRF do not have these problems and consequently, show a better performance.

**Figure 16:**
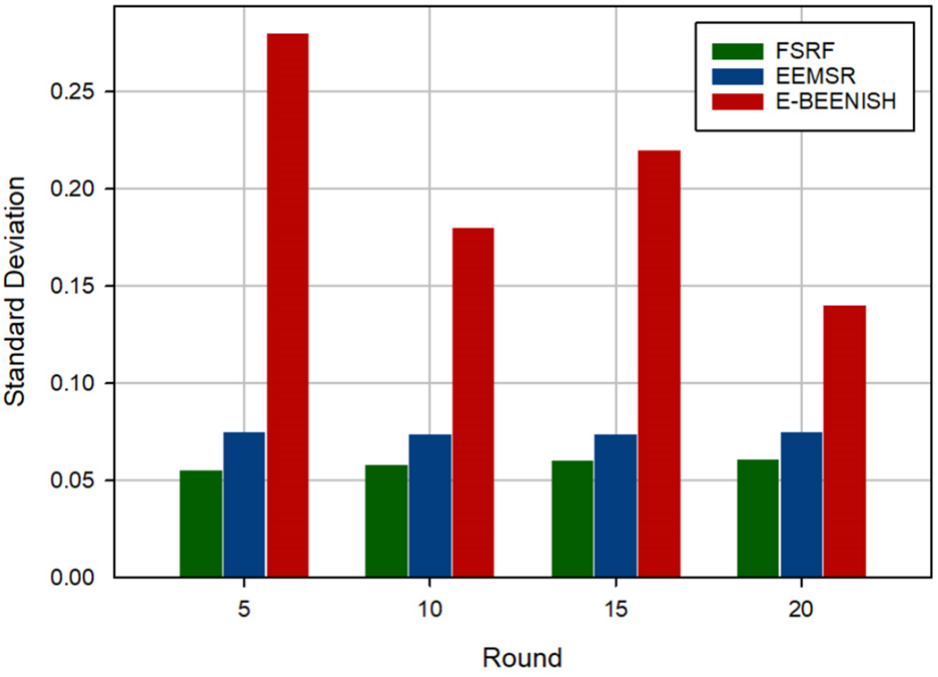
Evaluation of energy balance.

The fourth criterion for examining the performance of FSRF is to evaluate whether the energy is distributed between network nodes in a balanced manner. This criterion is determined by the standard deviation of the energy consumed in the nodes ($SD_{Energy}$). The results of this experiment are presented in Fig. 16. If $SD_{Energy}$ is near zero, it confirms that the energy is consumed in a balanced manner. In contrast, if $SD_{Energy}$ is close to one, it confirms that the energy is consumed in an imbalance manner. As Shown in Fig. 16, FSRF has the least $SD_{Energy}$, meaning that it can well balance the energy consumption between the network nodes. It reduced $SD_{Energy}$ by 21.48% and about 71.46% compared to EEMSR and E-BEENISH, respectively.

### Packet delivery rate

**Figure 17:**
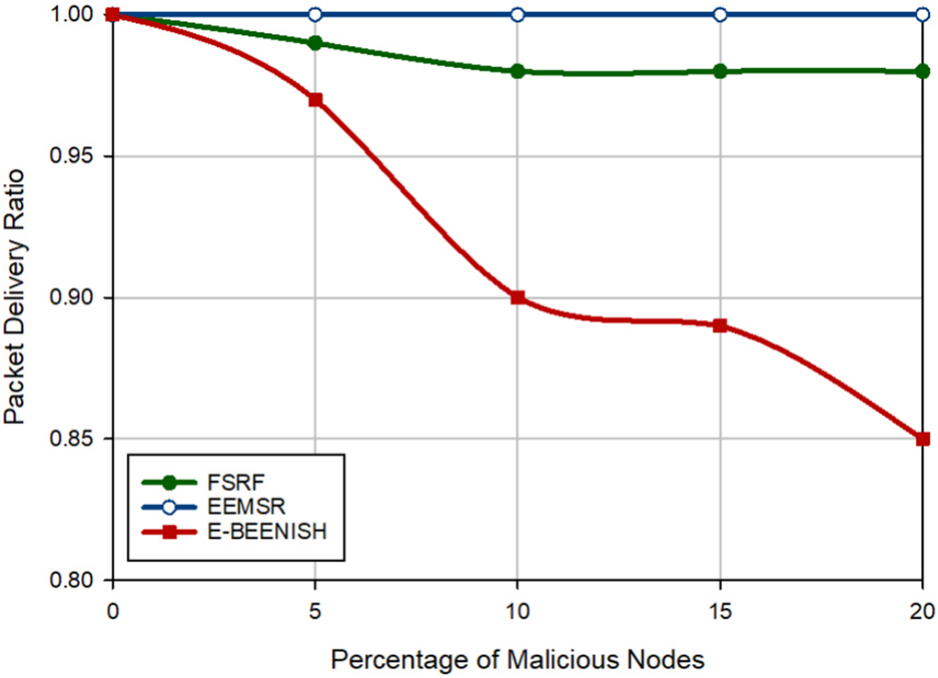
Evaluation of packet delivery rate.

**Figure 18:**
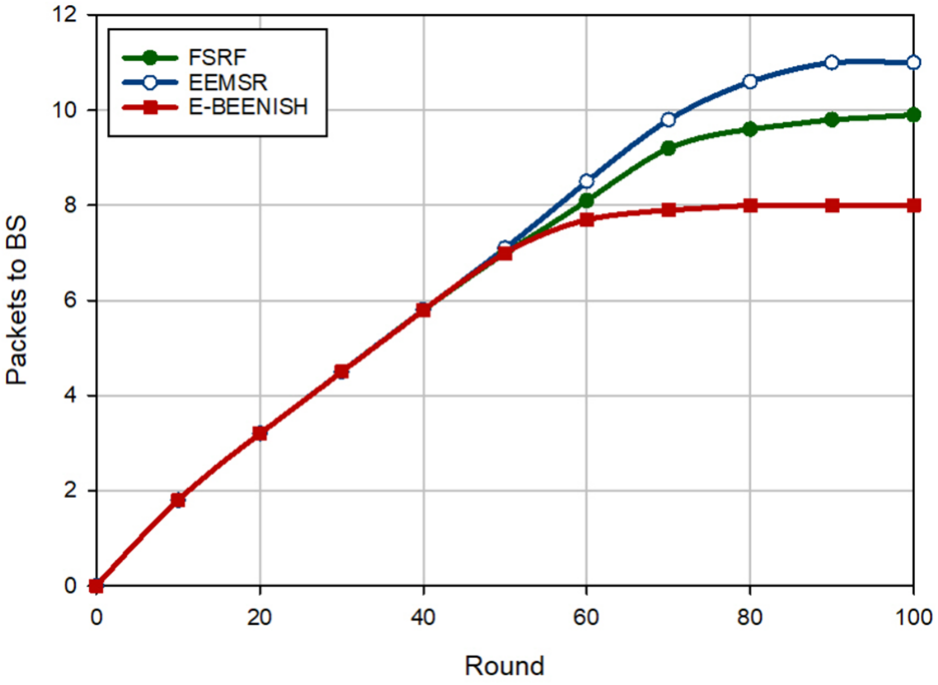
Evaluation of packets received by the base station.

The last criterion for the evaluation of FSRF is to investigate the packet delivery rate on the network. The results of this evaluation are shown in Figs. 17 and 18. Figure 17 presents the results of PDR according to the change in the percentage of hostile nodes in the network. FSRF has about less PDR (about 1.4%) than EEMSR because the trust mechanism designed in EEMSR is more powerful than that in FSRF. Furthermore, FSRF improves PDR (approximately 6.94%) compared to E-BEENISH because E-BEENISH has not paid attention to network security. As a result, PDR in E-BEENISH drops rapidly by increasing the percentage of hostile nodes on the network. In Fig. 18, the number of data packets delivered to the destination (i.e. BS) has been examined. According to this figure, FSRF has a lower PDR (approximately 6.01%) than EEMSR. However, PDR in FSRF has improved by approximately 11.16% compared to E-BEENISH. These results prove that EEMSR is more powerful than FSRF in terms of security. Whereas, FSRF works better than EEMSR in terms of energy efficiency, which is stated in Figs. 15 and 16.

## Conclusion

In this paper, a fuzzy secure hierarchical routing scheme based on the firefly algorithm (FSRF) was proposed for WSN-based IoT networks. This scheme seeks to achieve network security and energy efficiency. In FSRF, a fuzzy logic-based trust framework was presented to get the trust of nodes to detect and prevent various attacks such as black hole, flooding, wormhole, sinkhole, and Grey hole. Moreover, in FSRF, a FA-based clustering framework was designed to improve the energy consumption of nodes and network longevity. It comprises an objective function that considers trust amount, remaining energy, hops to BS, communication radius, and centrality. Finally, FSRF designs an inter-cluster routing framework to find reliable and energy-efficient paths on the network. Comparison of FSRF with EEMSR and E-BEENISH proved that the proposed method guarantees energy efficiency in the network because it improved network longevity by 10.34% and 56.35% and the energy stored in the nodes by 10.79% and 28.51% compared to EEMSR and E-BEENISH, respectively. However, FSRF is weaker than EEMSR in terms of security and has less PDR (almost 1.4%) than EEMSR. In future research directions, FSRF is evaluated under more scenarios to show its benefits and disadvantages. Moreover, we can test the robustness and efficiency of FSRF against various attacks. Furthermore, we will utilize new strategies like Q-learning and artificial neural networks (ANNs) to design robust trust frameworks to better separate abnormal nodes from normal nodes. For future work, this scheme can be improved for IoT networks with mobile nodes.

## Data Availability

All data generated or analyzed during this study are included in this published article.
